# Comparison of the 2022 world health organization classification and international consensus classification in myelodysplastic syndromes/neoplasms

**DOI:** 10.1038/s41408-024-01031-9

**Published:** 2024-04-09

**Authors:** Wan-Hsuan Lee, Chien-Chin Lin, Xavier Cheng-Hong Tsai, Feng-Ming Tien, Min-Yen Lo, Mei-Hsuan Tseng, Yuan-Yeh Kuo, Shan-Chi Yu, Ming-Chih Liu, Chang-Tsu Yuan, Yi-Tsung Yang, Ming-Kai Chuang, Bor-Sheng Ko, Jih-Luh Tang, Hsun-I Sun, Yi-Kuang Chuang, Hwei-Fang Tien, Hsin-An Hou, Wen-Chien Chou

**Affiliations:** 1 Divisions of Hematology, Department of Internal Medicine, National Taiwan University Hospital, Taipei, Taiwan; 2 Department of Internal Medicine, National Taiwan University Hospital, Hsin-Chu Branch, Hsinchu, Taiwan; 3 Graduate Institute of Clinical Medicine, College of Medicine, National Taiwan University, Taipei, Taiwan; 4 Department of Laboratory Medicine, National Taiwan University Hospital, Taipei, Taiwan; 5 Department of Medical Education and Research, National Taiwan University Hospital Yunlin Branch, Yunlin, Taiwan; 6 Department of Internal Medicine, National Taiwan University Hospital Yunlin Branch, Yunlin, Taiwan; 7 Tai-Chen Cell Therapy Center, National Taiwan University, Taipei, Taiwan; 8 Department of Pathology, National Taiwan University Hospital, Taipei, Taiwan; 9 Department of Pathology, National Taiwan University Hospital Cancer Center Branch, Taipei, Taiwan; 10 Department of Hematological Oncology, National Taiwan University Cancer Center, Taipei, Taiwan; 11 Department of Internal Medicine, Far-Eastern Memorial Hospital, New Taipei, Taiwan; 12 General Medicine, Department of Internal Medicine, National Taiwan University Hospital, Taipei, Taiwan

**Keywords:** Myelodysplastic syndrome, Genetic testing

## Abstract

In 2022, two novel classification systems for myelodysplastic syndromes/neoplasms (MDS) have been proposed: the International Consensus Classification (ICC) and the 2022 World Health Organization (WHO-2022) classification. These two contemporary systems exhibit numerous shared features but also diverge significantly in terminology and the definition of new entities. Thus, we retrospectively validated the ICC and WHO-2022 classification and found that both systems promoted efficient segregation of this heterogeneous disease. After examining the distinction between the two systems, we showed that a peripheral blood blast percentage ≥ 5% indicates adverse survival. Identifying MDS/acute myeloid leukemia with MDS-related gene mutations or cytogenetic abnormalities helps differentiate survival outcomes. In MDS, not otherwise specified patients, those diagnosed with hypoplastic MDS and single lineage dysplasia displayed a trend of superior survival compared to other low-risk MDS patients. Furthermore, the impact of bone marrow fibrosis on survival was less pronounced within the ICC framework. Allogeneic transplantation appears to improve outcomes for patients diagnosed with MDS with excess blasts in the ICC. Therefore, we proposed an integrated system that may lead to the accurate diagnosis and advancement of future research for MDS. Prospective studies are warranted to validate this refined classification.

## Introduction

Myelodysplastic neoplasms (MDS) are a diverse set of clonal myeloid neoplasms with dysregulated hematopoiesis, causing cytopenia and dysplastic hematopoietic cells. MDS is marked by recurrent chromosomal abnormalities, clinical and genetic diversity, and varying prognoses [1, 2]. The classification criteria for hematopoietic neoplasms, including MDS, were collated, beginning with the third edition of the World Health Organization (WHO) classification of hematologic malignancies in 2001 [3–5]. In 2016, the WHO, in collaboration with the Society for Hematopathology and the European Association for Hematopathology, published the revised fourth edition of the *WHO Classification of Tumours of Haematopoietic and Lymphoid Tissues* (WHO-2016) [6].

Recently, major advances in molecular technology and the development of next-generation sequencing (NGS) have deepened our understanding of MDS pathobiology [7, 8]. Revisions to the current classification system are needed for precise diagnosis, classification, and prognostication. Hence, the International Consensus Classification (ICC), developed with input from a global Clinical Advisory Committee of pathologists, hematologists, oncologists, and geneticists, was published in 2022 [9]. The ICC’s innovative changes in MDS include reclassifying MDS with 10–19% blasts as MDS/acute myeloid leukemia (AML), MDS with mutated *SF3B1* without excess blasts as MDS-*SF3B1* regardless of ring sideroblast count, and introducing novel molecular categories like myeloid neoplasms with mutated *TP53*, and MDS/AML with MDS-related gene mutations. Concurrently, the 5th edition of the WHO (WHO-2022) classification was released [10]. Emphasizing molecular features, tissue architecture, and histological appearance, WHO-2022 classification introduced new categories, including MDS with biallelic *TP53* inactivation (MDS-bi*TP53*), hypoplastic MDS (MDS-h), and MDS with fibrosis (MDS-f) [10]. These two systems exhibit several shared features but also differ significantly. The term MDS with increased blasts-2 remains in WHO-2022 classification but changes to MDS/AML in the ICC. Moreover, the ICC requires a 10% blast threshold to define AML with recurrent genetic abnormalities (excluding *BCR*::*ABL* and *TP53*), unlike WHO-2022, where most AML with defining genetic abnormalities can be diagnosed with increased blasts in peripheral blood (PB) or bone marrow (BM) (may be <20%). Additionally, a novel risk-scoring system, the Molecular IPSS (IPSS-M), combining clinical parameters, cytogenetic abnormalities, and somatic mutations of 31 genes, was established, stratifying MDS patients into six risk categories [11].

Few studies have concurrently explored the ICC and WHO-2022 classification within the IPSS-M context, evaluating their differences. This study retrospectively reviewed a cohort of 635 patients diagnosed with primary MDS using WHO-2016, WHO-2022, and ICC criteria. We elucidated differences in clinical characteristics, IPSS-M category distribution, genetic features, and outcomes among MDS subtypes per ICC and WHO-2022 classification and further compared these systems’ applicability in the IPSS-M framework.

## Materials and methods

A total of 635 patients with primary MDS whose BM samples were adequately cryopreserved for deep-targeted sequencing were included, and available data for the revised IPSS (IPSS-R) [12] and IPSS-M [11] risk assessments were obtained. Patients with prior chemotherapy/radiotherapy or hematologic malignancies were excluded, considering the distinct mutational profiles of primary and secondary MDS [13, 14]. BM cellularity and the grade of fibrosis were evaluated by reticulin staining and confirmed by pathologists. The Research Ethics Committee of the National Taiwan University Hospital approved this study (approval numbers: 201709072RINC, 202109078RINB, and 20220705RINB), and all participants provided written informed consent in accordance with the Declaration of Helsinki.

Cytogenetic analyses were performed and interpreted according to the International System for Human Cytogenetic Nomenclature [15, 16]. The TruSight myeloid sequencing panel (Illumina, San Diego, CS, USA) and HiSeq platform (Illumina, San Diego, CA, USA) were used to analyze alterations in 54 myeloid-neoplasm-related genes [11, 17, 18] (Supplementary Table 1). Five of the residual genes (*ETNK1, GNB1, NF1, PPM1D*, and *PRPF8*) defined by the IPSS-M model were not included and evaluation of *TP53* copy-neutral loss of heterozygosity were not avalible in the current study. Library preparation and sequencing were performed according to the manufacturer’s instructions. The median reading depth was 10550x. Somatic mutations were cataloged using cancer database v86, SNP database v151, ClinVar, PolyPhen-2, and SIFT algorithms for variant consequence evaluation. The variant analysis algorithm for diagnostics is previously described [19]. NGS limitations necessitated *FLT3*-ITD analysis via polymerase chain reaction (PCR) and fluorescence capillary electrophoresis and *KMT2A*-PTD analysis via PCR and Sanger sequencing [16, 20].

### Statistical analysis

The Mann–Whitney *U* test was used for continuous variables, and Fisher’s exact test or the χ^2^ test was used for discrete variables. The Kruskal-Wallis test was used to identify any significant disparities in medians among three or more groups. Leukemia-free survival (LFS) was defined as the interval between the date of diagnosis and the last follow-up, documented leukemic transformation, or death from any cause, whichever occurred first. Overall survival (OS) was defined as the interval between the date of diagnosis and the last follow-up or death from any cause, whichever occurred first. Survival curves were plotted using Kaplan-Meier analysis, and statistical significance was calculated using the log-rank test. The Cox proportional hazards model was used for univariable and multivariable analyses. Allogeneic hematopoietic stem cell transplantation (HSCT) was used as a time-dependent covariate [21]. All *P*-values were two-sided and considered statistically significant at *P* < 0.05. All analyses were performed using IBM SPSS Statistics v23 for Windows.

## Results

### Case allocation according to the ICC and 2022-WHO classification

**Fig. 1:**
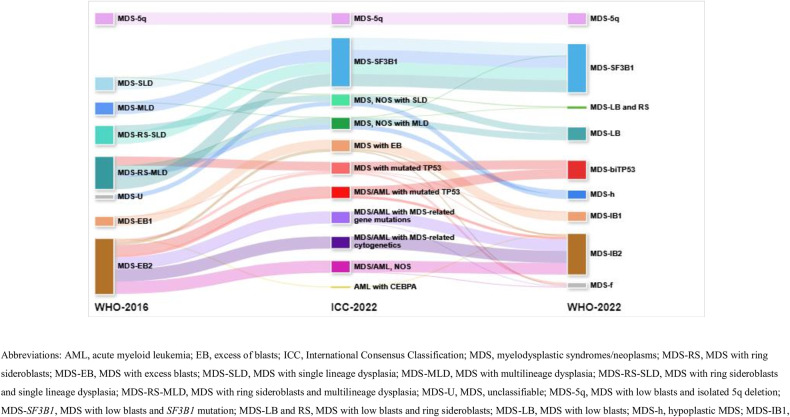
Case allocation from 2016 World Health Organization classification. Case allocation from 2016 World Health Organization classification to 2022 International Consensus Classification and 2022 World Health Organization classification of 635 patients with myelodysplastic syndromes/neoplasms.

The classification of the 635 patients diagnosed as having primary MDS based on the WHO-2016 classification and case allocation using the ICC and WHO-2022 criteria are presented in Table 1 and Fig. 1. In the ICC, 74.5% of patients remained in the MDS group, while 25.5% of patients were reclassified to the MDS/AML group. In the WHO-2016 classification, the MDS-5q subgroup was renamed to MDS with del(5q) in the ICC and MDS with low blasts and isolated 5q deletion in WHO-2022 classification. Nine (20.0%) patients in both the MDS with ring sideroblasts and single lineage dysplasia (MDS-RS-SLD) and MDS-RS and multilineage dysplasia (MDS-RS-MLD) subgroups, all with wild-type *SF3B1*, were distinguished from those with mutated *SF3B1* (Table 1).

**Table 1:** Case allocation from 2016 World Health Organization classification to 2022 International Consensus Classification and 2022 World Health Organization classification of 635 patients with myelodysplastic syndromes/neoplasms.

*AML* acute myeloid leukemia, *EB* excess of blasts, *ICC* International Consensus Classification, *MDS* myelodysplastic syndromes/neoplasms, *MDS-RS* MDS with ring sideroblasts, *MDS-EB* MDS with excess blasts, *MDS-SLD* MDS with single lineage dysplasia, *MDS-MLD* MDS with multilineage dysplasia, *MDS-RS-SLD* MDS with ring sideroblasts and single lineage dysplasia, *MDS-RS-MLD* MDS with ring sideroblasts and multilineage dysplasia, *MDS-U* MDS, unclassifiable, *MDS-5q* MDS with low blasts and isolated 5q deletion, *MDS-SF3B1* MDS with low blasts and *SF3B1* mutation, *MDS-LB and RS* MDS with low blasts and ring sideroblasts, *MDS-LB* MDS with low blasts, *MDS-h* hypoplastic MDS, *MDS-IB1* MDS with increased blasts-1, *MDS-IB2* MDS with increased blasts-2, *MDS-f* MDS with fibrosis, *MDS-biTP53* MDS with biallelic *TP53* inactivation, *NOS* not otherwise specified, *WHO* World Health Organization.

*MDS-related gene mutations: *ASXL1, BCOR, EZH2, RUNX1, SF3B1, SRSF2, STAG2, U2AF1*, or *ZRSR2*.

^†^MDS-related cytogenetic abnormalities: complex (≥3 clones) karyotype (in the absence of a *TP53* mutation), del(5q)/t(5q)/add(5q), -7/del(7q), +8, del(12p)/t(12p)/add(12p), i(17q), -17/add(17p) or del(17p), del(20q), and/or idic(X)(q13) clonal abnormalities.

Analysis of ICC vs. WHO-2022 classification differences showed that 39 (36.8%) and 55 (37.4%) individuals with MDS, not otherwise specified (NOS) with SLD or MLD, respectively, qualified for hypoplastic MDS (MDS-h) diagnosis. Meanwhile, nine (8.5%) in the MDS, NOS with SLD group, and seven (4.8%) in the MDS, NOS with MLD group (wild type *SF3B1*) were diagnosed as MDS with low blasts (LB) and RS in WHO-2022 classification (Table 1). Notably, three (2.0%) in the MDS, NOS with MLD group were diagnosed with MDS-*SF3B1* in WHO-2022 classification. Among these, two patients had *RUNX1* mutation and one had mutated *SF3B1* with variant allele frequency (VAF) <10%, excluding them from MDS-*SF3B1* in ICC. Thirteen (9.9%) diagnosed with MDS with excess blasts (EB) in ICC, having significant BM fibrosis, were classified as MDS-f in WHO-2022 classification. In WHO-2022 classification, PB blast percentage criteria remain (5–19% as increased blasts-2 [IB2]), but <10% is considered MDS with EB, not MDS/AML in ICC. Thus, nineteen (14.5%) with PB blast percentages of 5–9% were diagnosed as MDS-IB2 in WHO-2022 classification (Table 1).

Over 90% of patients with multi-hit *TP53* mutations exhibit complex karyotypes [22, 23]. However, the WHO-2022 criteria consider MDS-bi*TP53* only in cases with two or more *TP53* mutations or one mutation plus *TP53* copy number loss. Conversely, the ICC considers *TP53* mutation with complex karyotype as multi-hit *TP53* if *TP53* locus heterozygosity information is unavailable. Additionally, when PB or BM blast ranges from 10–19%, patients with not only multi-hit, but also single *TP53* mutation would be classified as MDS/AML with mutated *TP53*. Thus, 14 patients (23.3%) in the MDS or MDS/AML group with mutated *TP53* in the ICC were classified as MDS-IB or MDS-f in WHO-2022 classfication (Table 1, Fig. 1). Due to differing blast percentage criteria, three patients identified as AML with *CEBPA* mutation in the basic leucine zipper domain per ICC were placed in MDS-IB2 (14.5%, 15.2%, 19% blasts in BM) as per WHO-2022 criteria (Table 1, Fig. 1). The key diagnostic discrepancies between ICC and WHO-2022 classification due to different diagnostic criteria were summarized in Supplementary Table 2.

### Comparison of the demographic features of patients with different MDS subtypes

Clinical characteristics and genetic profiles of patients with various MDS subtypes per ICC/WHO-2022 classification are in Table 2, Supplementary Fig. 1, and Supplementary Table 3. In the ICC, all patients with mutated *TP53* had high- or very high-risk IPSS-R/IPSS-M, whereas only one patient with MDS-*SF3B1* had high-risk IPSS-M because of the presence of co-mutations including *CBL*, *GATA2*, and *CEBPA*. Moreover, MDS-EB subgroup patients were at higher risk according to IPSS-R or IPSS-M than MDS, NOS with SLD or MLD patients. All MDS/AML patients had intermediate to very high-risk IPSS-R. In the MDS/AML subgroup, 3.2% with MDS-related gene mutations and 5.0% with MDS/AML-NOS were moderate low-risk IPSS-M. MDS/AML patients with mutated *TP53* showed the highest incidence of very high-risk IPSS-R or IPSS-M. In the WHO-2022 classification, patients classified as having MDS-LB and RS had similar IPSS-R and IPSS-M risk stratification distributions to those with MDS-LB, which were distinct from those with MDS-*SF3B1*. Patients with MDS-*SF3B1* more frequently had very low, and low-risk IPSS-R (77.4%) and very low, low, and moderate low-risk IPSS-M (88.7%) than did patients with the other two subtypes (for IPSS-R: MDS-LB and RS vs. MDS-LB, 50.0% vs. 52.9%, *P* = 0.828, MDS-*SF3B1* vs. MDS-LB and RS or MDS-LB, both *P* < 0.05; for IPSS-M, MDS-LB and RS vs. MDS-LB, 37.5% vs. 63.6%, *P* = 0.058, MDS-*SF3B1* vs. MDS-LB and RS or MDS-LB, both *P* < 0.001; Table 2, Supplementary Figs. 1c and 1d). Patients with MDS-f exhibited similar BM blast percentage with MDS-IB1 (median blast 7% vs. 7%, *P* = 0.802), but significant less blast percentage than those with MDS-IB2 (median blast 7% vs. 13%, *P* < 0.001). The distributions of the IPSS-R and IPSS-M scores among patients with MDS-f were comparable to those among individuals with MDS-IB (Table 2, Supplementary Figs. 1c and 1d). Additionally, all patients with MDS-bi*TP53* had very high-risk IPSS-M (Table 2 and Supplemental Fig. 1d).

**Table 2:** Clinical characteristics of patients with myelodysplastic neoplasms, categorized by the 2022 World Health Organization classification.

Data are presented as n (%). *P* values of <0.05 are statistically significant.

*AML* acute myeloid leukemia, *ANC* absolute neutrophil count, *MDS* myelodysplastic neoplasms, *5q* MDS with low blasts and isolated 5q deletion, *biTP53* MDS with biallelic *TP53* inactivation, *f* MDS with fibrosis, *h* MDS hypoplastic, *Hb* hemoglobin, *HMA* hypomethylating agent, *HSCT* hematopoietic stem cell transplantation, *IB1* MDS with increased blasts-1, *IB2* MDS with increased blasts-2, *IPSS-R* revised international prognosis scoring system, *IPSS-M* molecular international prognosis scoring system, *LB* *MDS* with low blasts, *RS* ring sideroblasts, *SF3B1* MDS with low blasts and SF3B1 mutation.

*Median.

^†^Other treatment: include low-dose cytarabine, rabbit-derived anti-thymocyte globulin (rATG), cyclosporine, danazol, eltrombopag, erythropoietin-stimulating agents (ESA), thalidomide, steroid, venetoclax-based therapy and oral chemotherapy.

^§^Death within 3 months of diagnosis.

Fewer patients with MDS with mutated *TP53* (8.7%, ICC), MDS/AML with mutated *TP53* (8.1%, ICC), or MDS-bi*TP53* (10.9%, WHO-2022 criteria) underwent HSCT compared to those with other subtypes of MDS/AML, MDS-EB/IB, and MDS-f, despite being at a higher risk (Supplementary Table 3 and Table 2). This was attributed to a 22–43% early mortality rate at 3 months (Supplementary Table 3 and Table 2), no complete remission in patients before HSCT, and leukemic transformation within a median of 7.5 months post-MDS diagnosis, limiting transplantation feasibility in MDS-bi*TP53* patients.

### Comparison of the genetic profiles among patients with different MDS subtypes

In the ICC, patients with MDS, NOS with SLD, and those with MDS, NOS with MLD exhibited similar mutation profiles (Supplementary Table 4a, Supplementary Fig. 2). Patients with MDS-*SFB31* harbored distinct mutation patterns with higher frequencies of *DNMT3A* mutations (17% vs. 5%, *P* = 0.004) and *TET2* mutations (34% vs. 12%, *P* < 0.001) compared to those with MDS, NOS with SLD and MLD (Supplementary Table 4a). Moreover, patients with mutated *TP53* harbored a significantly higher frequency of *TET2* mutations (26% vs. 9%, *P* = 0.019) but a lower frequency of *ASXL1* (4% vs. 35%, *P* = 0.003) and *RUNX1* (0% vs. 22%, *P* = 0.008) mutations compared to the EB subgroup (Supplementary Table 4a).

On the other hand, based on the WHO-2022 criteria, patients with MDS-LB and MDS-LB and RS exhibited comparable mutation profiles (Supplementary Fig. 3), whereas patients with MDS-*SF3B1* had significantly different mutation patterns when compared to those of the former two subtypes. Patients with MDS-*SF3B1* had significantly more *DNMT3A* (16% vs. 6%, *P* = 0.029) and *TET2* mutations (32% vs. 14%, *P* = 0.004) but fewer *STAG2* mutation (2% vs. 9%, *P* = 0.068) than did those with MDS-LB, while fewer of them harbored *SRSF2* mutations when compared to those with MDS-LB and RS (3% vs. 25%, *P* = 0.014) (Supplementary Table 4b).

We evaluated the genetic profiles of the patients diagnosed with MDS-LB or MDS-h. With the exception of the *U2AF1* mutation (MDS-LB vs. MDS-h, 9% vs. 1%, *P* = 0.017), patients in these two groups exhibited similar molecular landscapes. Among the patients with MDS-IB1, MDS-IB2, or MDS-f, patients with MDS-IB2 exhibited higher frequencies of *NRAS* (9% vs. 2%, *P* = 0.019), *TET2* (19% vs. 10%, *P* = 0.059), and monoallelic *TP53* (7% vs. 1%, *P* = 0.031) mutations than did those with MDS-IB1. At the same time, patients with MDS-f exhibited lower incidences of *TET2* (0% vs. 19%, *P* = 0.020) and *STAG2* mutations (8% vs. 27%, *P* = 0.050) compared to those diagnosed with MDS-IB. Mutational profile analysis indicated that patients with biallelic *TP53* inactivation had specific genetic alterations, with different co-occurring patterns from those observed in patients with MDS-IB or MDS-f (Supplementary Fig. 3, and Supplementary Table 4b). Excluding *SF3B1* and *TP53*, the molecular features including the presence or absence of mutations and their VAF were similar across WHO-2022 categories (data not shown).

### Survival analysis and impact of HSCT

We performed pairwise comparisons of survivals among patients with each subtype of MDS, defined by the ICC and WHO-2022 classification, with the exclusion of the MDS-del(5q) subgroup, which comprised a very limited number of patients (*n* = 4). In the ICC, patients diagnosed with MDS, NOS with SLD or MLD had comparable prognoses (162.1 vs. 185.5 months, *P* = 0.274 for LFS; *P* = 0.269 for OS). Among the subtypes of MDS/AML, patients with MDS/AML-NOS had the best outcomes (MDS/AML-NOS vs. other MDS/AML, 14.5 vs. 8.4 months for LFS, *P* = 0.047; 28.1 vs. 14.2 months for OS, *P* = 0.008) (Supplementary Fig. 4). Furthermore, individuals carrying *TP53* mutations experienced the worst outcomes, regardless of their blast percentage (MDS with mutated *TP53* vs. MDS/AML with mutated *TP53*, 4.1 vs. 4.0 months for LFS, *P* = 0.396; 5.2 vs. 5.1 months for OS, *P* = 0.590) (Supplementary Fig. 4).

**Fig. 2:**
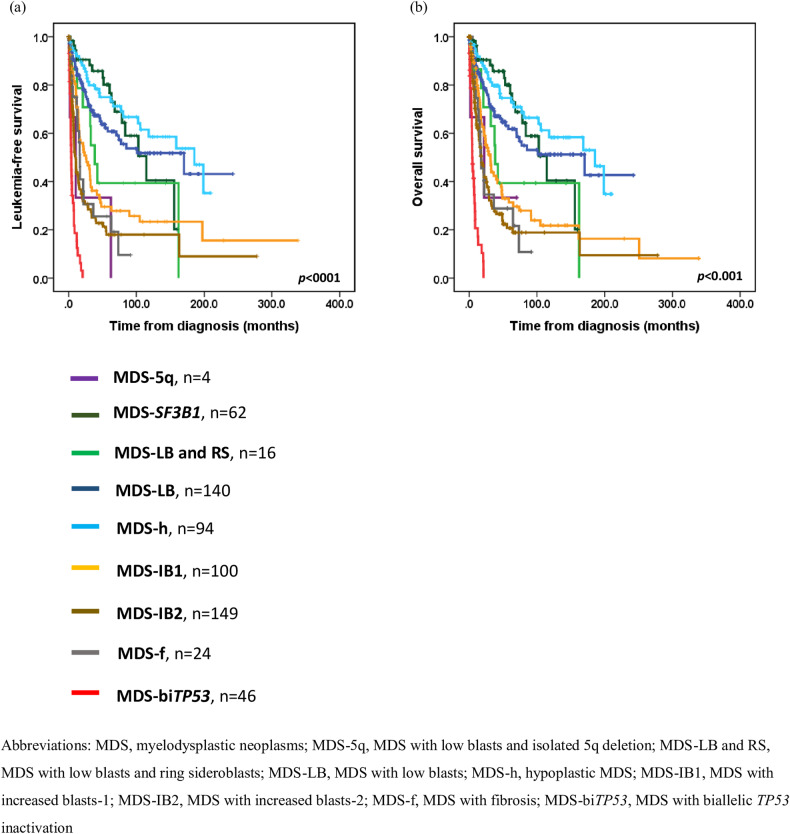
Kaplan-Meier curves for leukemia-free survival and overall survival in patients with myelodysplastic neoplasms based on the 2022 World Health Organization classification. **a** Leukemia-free survival for patients with myelodysplastic neoplasms. **b** Overall survival for patients with myelodysplastic neoplasms.

In the WHO-2022 classification, patients with MDS-h had a significantly longer median LFS and OS (185.5 months for both median LFS and OS) compared to those with MDS-LB and RS (38.3 months for median LFS [*P* = 0.013] and OS [*P* = 0.010]) but had a similar outcome to those with MDS-LB (170.2 months for median LFS [*P* = 0.152] and OS [*P* = 0.146]), and MDS-*SF3B1* (114.6 months for median LFS [*P* = 0.549] and OS [*P* = 0.528]) (Fig. 2). Notably, patients with bi*TP53* mutations had worse outcomes (median LFS: 3.9 months and median OS: 4.5 months, all *P* < 0.001) than did those with MDS-f (median LFS:16.6 months, and median OS:17.7 months), MDS-IB2 (median LFS:10.0 months, median OS:17.7 months), and MDS-IB1 (median LFS:25.6 months and median OS:31.4 months). Patients with MDS-f had similar survival to those with MDS-IB2, and patients with MDS-IB2 had a shorter LFS (*P* = 0.003) and OS (*P* = 0.027) than those with MDS-IB1 (Fig. 2). Additionally, we compared patients with different blast ranges in those with MDS-f. It showed that patients with PB blast 5–19%, or BM blast 10–19% (15.5 months for LFS, 16.3 months for OS) had a trend of shorter LFS (*P* = 0.087) and OS (*P* = 0.081) compared to those with less blasts (21.9 months for LFS, 73.3 months for OS; Supplementary Fig. 5).

Subgroup analysis of the impact of transplantation using time-dependent Cox regression analysis after adjusting for age revealed that in the ICC, HSCT could improve LFS (HR for LFS: 0.419, *P* = 0.019) in patients diagnosed with MDS with EB (Supplementary Table 5). Additionally, in the WHO-2022 classification, patients with MDS-IB1 may benefit from HSCT for LFS (HR for LFS: 0.450, *P* = 0.074); however, transplantation failed to improve the poor outcomes in patients with MDS-bi*TP53* (Supplementary Table 6).

### Subtypes analysis based on the interaction between the ICC and WHO-2022 classification

Based on the ICC, we further classified patients by the diagnostic criteria of the WHO-2022 classification and explored the survival and genetic differences between each subtype. In the group of patients with MDS, NOS with SLD (*n* = 106) or MLD (*n* = 147), patients could be classified into the subtypes of MDS-*SF3B1* (*n* = 3), MDS-LB and RS (*n* = 16), MDS-LB (*n* = 140), and MDS-h (*n* = 94), according to the WHO-2022 classification (Table 1). MDS-LB and RS were consolidated into the MDS-LB subgroup due to their comparable mutation profiles. Individuals diagnosed with MDS-h had a trend of better outcomes compared to those with MDS-LB (185.5 vs. 162.1 months, *P* = 0.075 for LFS; 185.5 vs. 101.1 months, *P* = 0.070 for OS) (Supplementary Fig. 6).

**Fig. 3:**
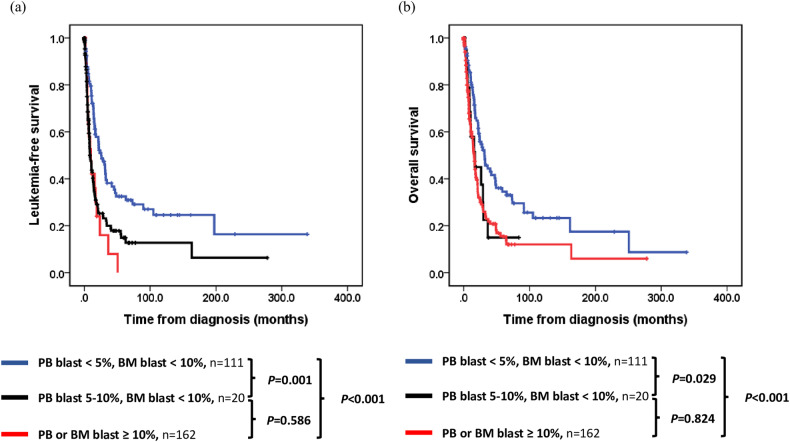
Kaplan-Meier curves for leukemia-free survival and overall survival in patients with myelodysplastic neoplasms, categorized by the blast percentage. **a** Leukemia-free survival for patients with myelodysplastic neoplasms. **b** Overall survival for patients with myelodysplastic neoplasms.

Patients diagnosed with MDS with EB (blast <10% in PB and BM) could be classified into the subtypes of MDS-IB1 (*n* = 99), MDS-IB2 (*n* = 19), and MDS-f (*n* = 13). Importantly, a blast percentage of 5–10% in PB implied shorter LFS (MDS-IB2 vs. MDS-IB1, 7.0 vs. 26.2 months, *P* = 0.004; MDS-IB2 vs. MDS-f, 7.0 vs. 21.9 months, *P* = 0.042). Patients with MDS-f did not show inferior outcome than those with MDS-IB1 (Supplementary Fig. 7). Moreover, patients with 5–10% blast in PB showed similarly poor outcomes as those classified as MDS/AML (blast percentage ≥10% in PB or BM; LFS 10.0 vs. 8.7 months, *P* = 0.586; OS 16.8 vs. 16.0 months, *P* = 0.824) (Fig. 3). Within the ICC framework, bone marrow fibrosis did not lead to further differentiation in survival outcomes across the genetic subgroups of MDS with mutated *TP53*, and MDS/AML with MDS-related gene mutations (Supplementary Figs. 8 and 9).

Alternatively, under the WHO-2022 classification, patients with MDS-LB could be reclassified into subtypes based on the ICC, including MDS, NOS with SLD (*n* = 58) or MDS, NOS with MLD (*n* = 82) (Supplementary Table 7). Patients with MLD, despite similar mutation profiles, had significantly lower white blood cell (2.9 vs. 3.7 × 10^9^ /L, *P* = 0.001), absolute neutrophil (1.5 vs. 2.2 × 10^9^ /L), and platelet counts (54 vs. 101 × 10^9^ /L, *P* = 0.006), with a trend towards shorter survivals than did SLD counterparts (77.3 vs. 170.2 months, *P* = 0.153 for LFS, *P* = 0.140 for OS). Furthermore, patients with MDS-h showed significantly longer survivals (median LFS and OS: 185.5 months; *P* = 0.037 for LFS, *P* = 0.034 for OS) compared to MLD, and similar outcomes (*P* = 0.828 for LFS; *P* = 0.824 for OS) to SLD (Supplemental Figure 10). Patients with MLD had a trend towards shorter survivals than did SLD counterparts.

For patients with biallelic *TP53* inactivation in the WHO-2022 criteria, MDS (*n* = 17) or MDS/AML (*n* = 29) with mutated *TP53* in the ICC (Supplementary Table 7) exhibited a similar prognosis (3.9 vs. 4.0 months for LFS, *P* = 0.211; 4.4 vs. 7.0 months for OS, *P* = 0.293) (Supplementary Fig. 11). Additionally, patients with MDS-IB2 could be classified as having MDS with EB (*n* = 19, peripheral blast 5–9%), MDS with mutated *TP53* (*n* = 1, single *TP5*3 mutation with VAF 41.2% and complex karyotype), MDS/AML with mutated *TP53* (*n* = 8), MDS/AML with MDS-related gene mutations (*n* = 87), MDS/AML with MDS-related cytogenetic abnormalities (*n* = 12), MDS/AML-NOS (*n* = 19), and AML with *CEBPA* (*n* = 3) (Supplementary Table 7). Patients with MDS/AML and MDS-related gene mutations had a higher risk under IPSS-R or IPSS-M than did those with MDS with EB (92.0% vs. 63.2% for high or very-high risk IPSS-R, *P* < 0.001; 95.4% vs. 73.6% for high or very-high risk IPSS-M, *P* = 0.002) and MDS/AML-NOS (95.4% vs. 73.7% for high or very-high risk IPSS-M, *P* = 0002).

Survival analysis among these patients showed that those with MDS/AML with mutated *TP53* (4.0 months for LFS, 4.1 months for OS) and with MDS-related cytogenetic abnormalities (10.6 months for LFS and OS) had the worst outcomes (MDS/AML with mutated TP53 vs. MDS/AML with MDS-related cytogenetic abnormalities, *P* = 0.399 for LFS, *P* = 0.131 for OS), followed by those with MDS-related gene mutations (vs. MDS/AML with mutated *TP53;* 10.7 months for LFS, *P* = 0.004; 20.7 months for OS, *P* < 0.001). Moreover, those with MDS/AML-NOS had the longest survivals (vs. MDS/AML with mutated *TP53;* 8.1 months for LFS, *P* = 0.052; 28.1 months for OS, *P* < 0.001) (Supplementary Fig. 12). In patients with MDS-f, survival differences were also noted among those with MDS with EB (*n* = 13, LFS 21.9 months, OS 22.2 months), MDS with mutated *TP53* (*n* = 4, LFS 5.2 months, OS 5.9 months), and MDS/AML with MDS-related gene mutations (*n* = 6, LFS 12.7 months, OS 13.9 months) when classified by the ICC (pairwise comparison *P* value all <0.05 for OS, Supplementary Fig. 13).

### Proposed integrated system based on the ICC and WHO-2022 classification

From our analysis, we propose: (1) MDS-h and MDS, NOS with SLD patients had better outcomes; (2) Patients with ≥5% blasts in PB show outcomes akin to MDS/AML, worse than do those with lower blasts; (3) When patients have a PB blast percentage ≥10%, not only multi-hit but also single-hit *TP53* mutations confer detrimental effects. Therefore, it is crucial to differentiate these patients from those with MDS with mutated *TP53*; (4) In MDS-IB2 patients, further distinction is needed for those with MDS-related gene mutations and cytogenetic abnormalities due to differing prognoses; (5) Within the ICC framework, BM fibrosis’ survival impact was less pronounced, prompting the refinement of the two-class system. MDS-h and MDS, NOS with SLD patients were segregated from other low blasts MDS and individuals with 5–10% PB blasts were shifted from MDS with EB to MDS/AML. Univariable analysis linked bedside IPSS-M, IPSS-R, older age, and male sex with poorer outcomes (Supplementary Table 8). Patients were categorized into five subgroups using the refined system. Multivariable analysis showed IPSS-M (*P* < 0.001), older age (*P* < 0.001), and the refined system (*P* < 0.001) as independent predictors of LFS and OS, with HSCT improving LFS (HR, 0.492, *P* = 0.001) (Table 3).

**Table 3:** Multivariable Cox regression analysis of the impact of different variables on the leukemia-free survival and overall survival of patients with myelodysplastic syndromes/neoplasms.

Note: Only 17 patients (2.7%) were categorized as IPSS-M very low risk and there was no inter-group difference between IPSS-M very low and low risk subgroups in both OS and LFS; accordingly, we put IPSS-M very low and low groups together.

*CI* confidence interval, *HR* hazard ratio, *HSCT* allogeneic hematopoietic stem cell transplantation, *h* hypoplastic, *IPSS-M* molecular international prognostic scoring system, *LFS* leukemia-free survival, *MDS* myelodysplastic syndromes/neoplasms, *OS* overall survival.

*P* values of <0.05 are statistically significant.

*As continuous variables analysis.

^†^Low-risk MDS included MDS with del(5q), MDS with mutated *SF3B1*, MDS, NOS with MLD.

^‡^MDS patients with EB and blast percentage < 5% in peripheral blood.

^§^MDS/AML with MDS-related gene mutations, MDS-related cytogenetic abnormalities, or not otherwise specified. Patients with MDS with EB and blast percentage ≥ 5% in peripheral blood were included in this group.

^¶^MDS or MDS/AML with mutated *TP53*, defined by International Consensus Classification.

## Discussion

In a cohort of 635 MDS patients, we retrospectively assessed the clinicopathological significance and prognostic implications of the ICC and WHO-2022 classification in the context of IPSS-M. Differences in clinical characteristics, genetic features, and outcomes among MDS subtypes based on these two novel systems were observed. Apart from the substitution of MDS-*SF3B1* for MDS-RS, additional innovative changes in the WHO-2022 criteria have included the introduction of MDS-bi*TP53*, MDS-f, and MDS-h. Moreover, the ICC highlights molecular features in diagnosis and classification, introducing categories like MDS with mutated *SF3B1* without excess blasts as MDS-*SF3B1* regardless of RS percentage and MDS or MDS/AML with mutated *TP53*. It also introduces the concept of MDS/AML with MDS-related gene mutations. These systems share many features yet differ in terminology and defining new entities [9, 10, 24]. The threshold for myelodysplastic features is set at 10% across all hematopoietic cell lineages in both systems. While SLD and MLD distinction remains in MDS, NOS subclassification in the ICC is optional in WHO-2022 criteria. Considering the heterogeneous results from previous studies [25, 26], we believe that further analysis is warranted to assess the survival impact of the lineage of dysplasia. Our study also revealed distinct features in patients with MDS-*SF3B1* compared to those with MDS-LB and RS, and MDS-LB, the latter two subtypes showing similar mutational landscapes.

Additionally, blast percentage criteria variances led to diagnostic discrepancies between MDS and AML, such as AML with *CEBPA* mutations (≥10% blasts in ICC; ≥20% in WHO-2022 criteria) or AML with mutated *NPM1* (≥10% blasts in ICC; increased blasts percentage in WHO-2022 criteria). The term MDS with IB2 is retained in the WHO-2022 criteria, whereas it is modified to MDS/AML in the ICC to emphasize the continuum spectrum between MDS and AML. In the group of MDS/AML, patients could be further classified according to genetic profiles (*TP53* mutation or MDS-related gene mutations) and cytogenetic abnormalities. Exploring these subtypes, we found PB blast percentage ≥ 5% linked to adverse outcomes, classifying it under the MDS/AML subtype. Identifying MDS/AML with MDS-related gene mutations or cytogenetic abnormalities aids in distinguishing patients’ survival rates.

MDS-h, a new entity in the WHO-2022 classification but not in ICC, comprises 10–27% of all MDS cases [27–29]. It is frequently observed in Asian MDS cohort or children cases [30, 31]. The different reported prevalence may result from (1) Variation in the criteria used to define hypocellular bone marrow across different studies; (2) Differences in patient enrollment criteria, including whether the study included de novo cases only or both de novo and secondary cases, as well as differences between French-American-British- and WHO-defined cohorts; (3) Variances in genetic and environmental backgrounds among the study populations; (4) The possibility of inadvertently including patients with aplastic anemia in the MDS-h cohort. In our study, 14.8% of patients were qualified for the diagnosis of MDS-h based on WHO-2022 definition (hypocellular marrow: <30% of normal cellularity in patients younger than 70 years and <20% in patients aged 70 and older). MDS-h patients show activated immune system features, especially effector T cells targeting hematopoietic stem and progenitor cells [27, 32]. They typically have severe cytopenia, fewer somatic mutations [28], higher immunosuppressive therapy response [33], and better outcomes with regards to the low-risk IPSS-R [34–36]. In our study, MDS, NOS patients with MDS-h showed a trend of improved survival compared to other low-risk MDS cases. Recognizing these patients aids in enhancing patient care through tailored treatment strategies.

BM fibrosis is correlated with higher white blood cell counts, BM blast percentages, and more pronounced dysmegakaryocytopoiesis in MDS. It is also associated with mutations in the *TP53*, *SETBP1*, and *JAK2* genes [37, 38]. Additionally, several studies have recognized fibrosis as an independent factor for poor prognosis [37, 39–42]. We found that in the patients with increased blasts, BM fibrosis adversely affected outcomes in the WHO-2022 classification, and patients with MDS-f had fewer *STAG2* and *TET2* mutations. However, the negative impact of BM fibrosis appeared to be less prominent within the framework of the ICC.

Up to 10% of patients with primary MDS have mutations in *TP53*, which results in a heightened risk of AML transformation and dismal outcomes [43], particularly in the setting of multiple hits [23]. Only patients with multiple hits displayed specific associations with complex karyotypes, a few co-occurring mutations, high-risk presentations, and short survival [23]. Furthermore, monoallelic patients did not differ from *TP53* wild-type patients in terms of outcome or response to therapy [23]. In this study, MDS-bi*TP53* patients had the shortest survival, with a distinct mutational landscape compared to MDS-IB or MDS-f patients (Supplementary Fig. 3 and Supplementary Table 4b). MDS or MDS/AML patients with *TP53* mutations defined by the ICC had unfavorable prognoses. Patients with a single *TP53* mutation and complex karyotype showed outcomes similar to those with multiple *TP53* mutations, justifying classifying these cases into this entity. Cox regression analysis revealed HSCT did not improve outcomes in these patients, as per either ICC or 2022-WHO classification (Supplementary Table 5, 6), consistent with prior reports [44–46].

Through an expanded analysis of 7,017 patients on behalf of the International Consortium for MDS [47], Komrokji et al. documented that genetically defined entities (*SF3B1*, del5q, and bi*TP53*) were unique, and the survival of patients with LB and RS (wild-type *SF3B1*) was similar to that of patients with MDS-LB. Our results are consistent with these findings. Although MDS-IB1 patients had longer OS, their LFS was comparable to MDS-IB2 in the Moffitt Cancer Center cohort. Survival analysis of our and the GenoMed4all cohort showed significant LFS and OS differences between subtypes [47]. Thus, the optimal cutoff value for the blast percentage requires further investigation. A unified classification system that included MDS-5q, MDS-*SF3B1*, MDS-h, MDS-SLD, MDS-MLD, MDS-LB, MDS-bi*TP53*, MDS-f, and MDS-EB was proposed by the Moffitt Cancer Center [48]. According to our analysis, we tried to refine and tie two classification systems together. We posited that MDS-h subtype, distinguishable from other low blasts MDS, should be separately recognized. Patients with PB blast ≥5% should be identified due to poorer outcomes.

The study’s limitations include its retrospective nature, limited case number, exclusion of five of the residual genes defined by the IPSS-M model, unavailability of *TP53* copy-neutral loss of heterozygosity, and heterogeneity in treatment regimens, though most high-risk MDS patients received hypomethylating agents or transplantation. The analysis for the impact of HSCT might be compromised due to patients’ comorbidities, pre-transplant treatments, and transplant modalities, et al. Prospective studies are needed to validate the refined MDS classification and assess HSCT’s impact on high-risk MDS patient outcomes.

In conclusion, the ICC and WHO-2022 classification effectively segregate this heterogeneous disease. However, coexisting diagnostic standards challenge clinicians in treatment and diagnosis and hinder clinical trials and research progress. Therefore, we propose an integrated classification system for accurate MDS diagnosis and effective risk-adapted treatment.

## Supplementary information

Supplemental material

## Data Availability

The datasets generated during and/or analyzed during this study are available from the corresponding author upon reasonable request.
