# Supplementary material for: Comparison of the 2022 world health organization classification and international consensus classification in myelodysplastic syndromes/neoplasms
Source: Blood Cancer J. 2024 Apr 9;14(1):57. doi: 10.1038/s41408-024-01031-9 (PMC11004131; doi:10.1038/s41408-024-01031-9)
Supplement: Supplementary file 1 — Supplemental material [file 41408_2024_1031_MOESM1_ESM.docx]

**Supplemental Table 1. List of 54 myeloid neoplasm-relevant genes studied in targeted NGS sequencing**

| **Gene name** | **Target region (exon)** | **Gene name** | **Target region (exon)** |
| --- | --- | --- | --- |
| ***ABL*** | 4-6 | ***JAK3*** | 13 |
| ***ASXL1*** | 12 | ***KDM6A*** | full |
| ***ATRX*** | 8-10, 17-31 | ***KIT*** | 2, 8-11, 13, 17 |
| ***BCOR*** | full | ***KRAS*** | 2, 3 |
| ***BCORL1*** | full | ***KMT2A*** | 5-8 |
| ***BRAF*** | 15 | ***MPL*** | 10 |
| ***CALR*** | 9 | ***MYD88*** | 3-5 |
| ***CBL*** | 8, 9 | ***NOTCH1*** | 26-28, 34 |
| ***CBLB*** | 9, 10 | ***NPM1*** | 12 |
| ***CBLC*** | 9, 10 | ***NRAS*** | 2, 3 |
| ***CDKN2A*** | full | ***PDGFRA*** | 12, 14, 18 |
| ***CEBPA*** | full | ***PHF6*** | Full |
| ***CSF3R*** | 14-17 | ***PTEN*** | 5, 7 |
| ***CUX1*** | full | ***PTPN11*** | 3, 13 |
| ***DNMT3A*** | full | ***RAD21*** | Full |
| ***ETV6*** | full | ***RUNX1*** | Full |
| ***EZH2*** | full | ***SETBP1*** | 4 (partial) |
| ***FBXW7*** | 9-11 | ***SF3B1*** | 13-16 |
| ***FLT3*** | 14, 15, 20 | ***SMC1A*** | 2, 11, 16, 17 |
| ***GATA1*** | 2 | ***SMC3*** | 10, 13, 19, 23, 25, 28 |
| ***GATA2*** | 2-6 | ***SRSF2*** | 1 |
| ***GNAS*** | 8, 9 | ***STAG2*** | full |
| ***HRAS*** | 2, 3 | ***TET2*** | 3-11 |
| ***IDH1*** | 4 | ***TP53*** | 2-11 |
| ***IDH2*** | 4 | ***U2AF1*** | 2, 6 |
| ***IKZF1*** | full | ***WT1*** | 7, 9 |
| ***JAK2*** | 12, 14 | ***ZRSR2*** | full |

**Supplemental Table 2. Diagnostic discrepancies between International Consensus Classification and 2022 World Health Organization classification for myelodysplastic syndromes/neoplasms due to different diagnostic criteria.**

| **Categories** | **WHO-2022 classification** | | **ICC** | |
| --- | --- | --- | --- | --- |
|  | Diagnostic criteria | Number | Diagnostic criteria | Number |
| MDS-*SF3B1* | 1. *SF3B1* VAF **≥5%** 2. Absence of multi-hit *TP53* mutation 3. Absence of 5q deletion, monosomy 7/7q deletion, or complex karyotype. | 62 | 1. *SF3B1* VAF **≥10%** 2. Absence of multi-hit *TP53* mutation and ***RUNX1*** mutation 3. Absence of isolated del(5q), - 7/del(7q), **abn3q26.2**, or complex | 59 |
| MDS with mutated *TP53* or MDS-bi*TP53* | 1. **No blast percentage criteria** 2. Two distinct *TP53* mutations 3. Single *TP53* mutation with evidence of TP53 copy neutral LOH. 4. TP53 VAF >49% as presumptive (not definitive) of copy neutral LOH 5. **No VAF requirement** | 46 | 1. **PB or BM blast <10%** 2. Two distinct *TP53* mutations (each **VAF >10%**) 3. Single TP53 mutation with either 1) 17p deletion on cytogenetics; 2) VAF of >50%; 3) Copy-neutral LOH at the 17p *TP53* locus. 4. ***TP53* mutation (VAF >10%) and complex karyotype often with loss of 17p** | 23 |
| MDS/AML with mutated *TP53* | Not an entity | 0 | 1. **PB or BM blast 10-19%** 2. **Any somatic *TP53* mutation (VAF >10%)** | 37 |
| Increased blasts^*^ | PB blast 2-19% or BM blast 5-19% with fibrosis: **MDS-f** | 24 | PB or BM blast 10-19% with **MDS-related gene mutations** | 93 |
|  | **PB blast 5-19%** or BM blast 10-19%: MDS-IB2 | 100 | PB or BM blast 10-19% with **MDS-related cytogenetic abnormalities** | 12 |
|  | **PB blast 2-4%** or BM blast 5-9%: MDS-IB1 | 149 | NOS | 20 |

Note: Key differences of diagnostic criteria between WHO-2022 classification and ICC has been marked in bold.

^*^Listed based on diagnostic hierarchy

Abbreviation: AML, acute myeloid leukemia; BM, bone marrow; EB, excess blasts; ICC, International Consensus Classification; LOH, loss of heterozygosity; MDS, myelodysplastic syndromes/neoplasms; MDS-bi*TP53*, MDS with biallelic *TP53* inactivation; MDS-f, MDS with fibrosis; MDS-IB1, MDS with increased blasts-1; MDS-IB2, MDS with increased blasts-2; PB, peripheral blood; RS, ring sideroblasts; VAF, variant allele frequency; WHO, World Health Organization.

**Supplemental Table 3a. Clinical characteristics of patients with myelodysplastic syndromes, categorized by the 2022 International Consensus Classification**

| Variables | Del(5q)  (n=4) | Mutated *SF3B1*  (n=59) | NOS,  SLD  (n=106) | NOS,  MLD  (n=147) | EB  (n=131) | Mutated *TP53*  (n=23) | *P* value |
| --- | --- | --- | --- | --- | --- | --- | --- |
| Sex |  |  |  |  |  |  | **0.076** |
| Female | 3 (75.0) | 17 (28.8) | 51 (46.4) | 49 (32.7) | 51 (38.9) | 6 (26.1) |  |
| Male | 1 (25.0) | 42 (71.2) | 59 (53.6) | 101 (67.3) | 80 (61.1) | 17 (73.9) |  |
| Age* | 71 (70-75) | 70 (27-91) | 67 (21-95) | 63 (18-94) | 67 (23-94) | 71 (46-91) | **0.074** |
| Laboratory data* |  |  |  |  |  |  |  |
| WBC, ×10^9^ /L | 3.5 (2.0-4.2) | 3.9 (1.8-7.9) | 3.9 (1.1-26.3) | 2.8 (0.8-15.8) | 3.1 (0.7-32.4) | 3.2 (1.1-12.2) | **<0.001** |
| ANC, ×10^9^ /L | 1.7 (0.5-2.4) | 2.3 (0.4-5.1) | 2.2 (0.1-12.3) | 1.4 (0-10.8) | 1.4 (0-23.5) | 1.5 (0.2-3.5) | **<0.001** |
| Hb, g/dL | 5.7 (3.5-7.5) | 8.1 (5.4-11.7) | 7.9 (3.4-14.4) | 8.3 (4.1-17.1) | 8.3 (3.2-13.7) | 7.1 (4.4-9.7) | **0.035** |
| Platelet, ×10^9^ /L | 214 (82-427) | 194 (6-471) | 106 (4-607) | 52 (3-405) | 71 (1-721) | 65 (1-133) | **<0.001** |
| BM blast (%) | 2.4 (0-4.1) | 1.6 (0-4.6) | 1.5 (0-4.8) | 2.0 (0-4.9) | 6.5 (0.6-9.8) | 7.4 (1.4-9.5) | **<0.001** |
| PB blast (%) | 0 (0) | 0 (0) | 0 (0) | 0 (0-1) | 0 (0-9) | 2 (0-9) | **<0.001** |
| IPSS-R |  |  |  |  |  |  | **<0.001** |
| Very low | 0 (0.0) | 7 (11.9) | 9 (8.5) | 6 (4.1) | 0 (0.0) | 0 (0.0) | **0.001** |
| Low | 1 (25.0) | 40 (67.8) | 52 (49.1) | 64 (43.5) | 12 (9.2) | 0 (0.0) | **<0.001** |
| Intermediate | 3 (75.0) | 12 (20.3) | 38 (35.8) | 55 (37.4) | 47 (35.9) | 0 (0.0) | **0.001** |
| High | 0 (0.0) | 0 (0.0) | 5 (4.7) | 17 (11.6) | 50 (38.2) | 2 (8.7) | **<0.001** |
| Very high | 0 (0.0) | 0 (0.0) | 2 (1.9) | 5 (3.4) | 22 (16.8) | 21 (91.3) | **<0.001** |
| IPSS-M |  |  |  |  |  |  | **<0.001** |
| Very low | 0 (0.0) | 2 (3.4) | 5 (4.7) | 9 (6.1) | 2 (1.5) | 0 (0.0) | 0.412 |
| Low | 0 (0.0) | 39 (66.1) | 41 (38.7) | 44 (29.9) | 8 (6.1) | 0 (0.0) | **<0.001** |
| Moderate low | 0 (0.0) | 13 (22.0) | 29 (27.4) | 31 (21.1) | 16 (12.2) | 0 (0.0) | **0.009** |
| Moderate high | 2 (50.0) | 4 (6.8) | 19 (17.9) | 31 (21.1) | 26 (19.8) | 0 (0.0) | **0.025** |
| High | 1 (25.0) | 1 (1.7) | 10 (9.4) | 23 (15.6) | 50 (38.2) | 0 (0.0) | **<0.001** |
| Very high | 1 (25.0) | 0 (0.0) | 2 (1.9) | 9 (6.1) | 29 (22.1) | 23 (100) | **<0.001** |
| Treatment |  |  |  |  |  |  |  |
| HMA | 1 (25.0) | 4 (6.8) | 2 (1.9) | 9 (6.1) | 54 (41.2) | 11 (47.8) | **<0.001** |
| Intensive chemotherapy | 0 (0.0) | 1 (1.8) | 1 (1.0) | 1 (0.7) | 6 (4.6) | 0 (0.0) | 0.204 |
| Clinical trial | 0 (0.0) | 0 (0.0) | 6 (5.8) | 5 (3.4) | 7 (5.4) | 1 (4.5) | 0.600 |
| HSCT | 0 (0.0) | 6 (10.2) | 3 (2.8) | 16 (10.9) | 39 (29.8) | 2 (8.7) | **<0.001** |
| Supportive care | 3 (75.0) | 35 (62.5) | 63 (61.2) | 88 (59.9) | 39 (30.0) | 9 (40.9) | **<0.001** |
| Other treatment^†^ | 0 (0.0) | 16 (28.6) | 31 (30.1) | 43 (29.3) | 22 (16.9) | 2 (9.1) | **0.033** |
| AML transformation | 2 (50.0) | 3 (5.1) | 8 (7.5) | 13 (8.8) | 41 (31.3) | 11 (47.8) | **<0.001** |
| Death | 2 (50.0) | 15 (25.4) | 35 (33.0) | 55 (37.4) | 80 (61.1) | 19 (82.6) | **<0.001** |
| Early mortality^§^ | 2 (50.0) | 6 (10.2) | 7 (6.6) | 16 (10.9) | 13 (9.9) | 5 (21.7) | **0.036** |

No patients qualify for the diagnosis of MDS, NOS without dysplasia. *P* values of <0.05 are statistically significant

*Median (range).

^†^Other treatment: include low-dose cytarabine, rabbit-derived anti-thymocyte globulin (rATG), cyclosporine, danazol, eltrombopag, erythropoietin-stimulating agents (ESA), thalidomide, steroid, venetoclax-based therapy and oral chemotherapy.

^§^Death within 3 months of diagnosis.

Abbreviations: ANC, absolute neutrophil count; AML, acute myeloid leukemia; BM, bone marrow; del, deletion; EB, excess blasts; Hb, hemoglobin; HMA, hypomethylation agent; HSCT, allogeneic hematopoietic stem cell transplantation; IPSS-R, revised international prognosis scoring system; IPSS-M, Molecular International Prognosis Scoring System; MLD, multilineage dysplasia; NOS, not otherwise specified; PB, peripheral blood; SLD, single lineage dysplasia

**Supplemental Table 3b. Clinical characteristics of patients with myelodysplastic syndromes/acute myeloid leukemia, categorized by the 2022 International Consensus Classification**

| Variables | Mutated *TP53*  (n=37) | MDS-related gene mutations  (n=93) | MDS-related cytogenetics  (n=12) | NOS  (n=20) | *P* value |
| --- | --- | --- | --- | --- | --- |
| Sex |  |  |  |  | 0.231 |
| Female | 16 (43.2) | 26 (28.0) | 6 (50.0) | 7 (35.0) |  |
| Male | 21 (56.8) | 67 (72.0) | 6 (50.0) | 13 (65.0) |  |
| Age* | 68 (27-85) | 67 (26-91) | 65 (46-86) | 59 (30-76) | **0.043** |
| Laboratory data* |  |  |  |  |  |
| WBC, ×10^9^ /L | 3.3 (0.6-17.5) | 3.1 (0.6-30.3) | 2.8 (0.9-11.1) | 2.3 (0.8-14.9) | 0.646 |
| ANC, ×10^9^ /L | 1.1 (0.1-11.7) | 1.1 (0.1-13.9) | 1.1 (0.4-3.8) | 1.3 (0-6.5) | 0.788 |
| Hb, g/dL | 7.9 (5.3-11.9) | 8.2 (2.6-14.6) | 8.1 (6.9-11.7) | 8.3 (55.7-12.7) | 0.900 |
| Platelet, ×10^9^ /L | 46 (7-460) | 78 (4-931) | 83 (8-206) | 96 (14-295) | 0.133 |
| BM blast (%) | 14.2 (10.2-19.5) | 13.0 (5.0-19.4) | 13.6 (10.2-17.2) | 12.0 (10.0-18.6) | 0.286 |
| PB blast (%) | 2 (0-17) | 1 (0.0-19) | 1 (0-16) | 2 (0-18) | **0.018** |
| IPSS-R |  |  |  |  | **<0.001** |
| Very low | 0 (0.0) | 0 (0.0) | 0 (0.0) | 0 (0.0) | **-** |
| Low | 0 (0.0) | 0 (0.0) | 0 (0.0) | 0 (0.0) | **-** |
| Intermediate | 0 (0.0) | 7 (7.5) | 0 (0.0) | 4 (20.0) | **0.027** |
| High | 0 (0.0) | 51 (54.8) | 3 (25.0) | 8 (40.0) | **<0.001** |
| Very high | 37 (100) | 35 (37.6) | 9 (75.0) | 8 (40.0) | **<0.001** |
| IPSS-M |  |  |  |  | **<0.001** |
| Very low | 0 (0.0) | 0 (0.0) | 0 (0.0) | 0 (0.0) | **-** |
| Low | 0 (0.0) | 0 (0.0) | 0 (0.0) | 0 (0.0) | **-** |
| Moderate low | 0 (0.0) | 3 (3.2) | 0 (0.0) | 1 (5.0) | 0.574 |
| Moderate high | 0 (0.0) | 2 (2.2) | 1 (8.3) | 5 (25.0) | **<0.001** |
| High | 0 (0.0) | 18 (19.4) | 3 (25.0) | 8 (40.0) | **0.002** |
| Very high | 37 (100) | 70 (75.3) | 8 (66.7) | 6 (30.0) | **<0.001** |
| Treatment |  |  |  |  |  |
| HMA | 16 (43.2) | 41 (44.1) | 8 (66.7) | 5 (25.0) | 0.143 |
| Intensive chemotherapy | 1 (2.7) | 5 (5.4) | 0 (0.0) | 3 (15.0) | 0.196 |
| Clinical trial | 2 (5.4) | 5 (5.4) | 0 (0.0) | 1 (5.0) | 0.878 |
| HSCT | 3 (8.1) | 21 (22.6) | 2 (16.7) | 6 (30.0) | 0.171 |
| Supportive care | 14 (37.8) | 24 (25.8) | 3 (25.0) | 6 (30.0) | 0.579 |
| Other treatment^†^ | 5 (13.5) | 14 (15.1) | 0 (0.0) | 5 (25.0) | 0.286 |
| AML transformation | 13 (35.1) | 43 (46.2) | 3 (25.0) | 10 (50.0) | 0.346 |
| Death | 26 (70.3) | 62 (66.7) | 8 (66.7) | 9 (45.0) | 0.253 |
| Early mortality^§^ | 16 (43.2) | 10 (10.8) | 4 (33.3) | 1 (5.0) | **<0.001** |

*P* values of <0.05 are statistically significant

*Median (range).

^†^Other treatment: include low-dose cytarabine, rabbit-derived anti-thymocyte globulin (rATG), cyclosporine, danazol, eltrombopag, erythropoietin-stimulating agents (ESA), thalidomide, steroid, venetoclax-based therapy and oral chemotherapy.

^§^Death within 3 months of diagnosis.

Abbreviations: ANC, absolute neutrophil count; AML, acute myeloid leukemia; BM, bone marrow; Hb, hemoglobin; HMA, hypomethylation agent; HSCT, allogeneic hematopoietic stem cell transplantation; IPSS-R, revised international prognosis scoring system; IPSS-M, Molecular International Prognosis Scoring System; NOS, not otherwise specified; PB, peripheral blood

**Supplemental Table 4a. Comparison of gene alterations, grouped by functional categories, among patients with different subtypes of myelodysplastic syndromes, categorized by the 2022 International Consensus Classification**

| Genes | Del(5q)  (n=4) | Mutated *SF3B1*  (n=59) | NOS,  SLD  (n=106) | NOS,  MLD  (n=147) | EB  (n=131) | Mutated *TP53*  (n=23) | *P* value |
| --- | --- | --- | --- | --- | --- | --- | --- |
| Epigenetics modifiers | 3 (75) | 30 (51) | 27 (26) | 46 (31) | 77 (59) | 9 (39) | **<0.001** |
| DNA methylation | 2 (50) | 26 (44) | 16 (15) | 26 (18) | 34 (26) | 7 (30) | **<0.001** |
| *DNMT3A* | 1 (25) | 10 (17) | 5 (5) | 7 (5) | 14 (11) | 2 (9) | **0.021** |
| *TET2* | 1 (25) | 20 (34) | 11 (10) | 18 (12) | 12 (9) | 6 (26) | **<0.001** |
| *IDH1* | 0 (0) | 0 (0) | 0 (0) | 0 (0) | 0 (0) | 0 (0) | - |
| *IDH2* | 0 (0) | 1 (2) | 3 (3) | 2 (1) | 7 (5) | 0 (0) | 0.434 |
| *WT1* | 0 (0) | 0 (0) | 0 (0) | 2 (1) | 5 (4) | 0 (0) | 0.236 |
| Chromatin modifiers | 1 (25) | 7 (12) | 17 (16) | 29 (20) | 62 (47) | 2 (9) | **<0.001** |
| *ASXL1* | 0 (0) | 5 (9) | 12 (11) | 19 (13) | 46 (35) | 1 (4) | **<0.001** |
| *EZH2* | 1 (25) | 1 (2) | 1 (1) | 4 (3) | 10 (8) | 0 (0) | **0.024** |
| *MLL* | 0 (0) | 1 (2) | 0 (0) | 3 (2) | 1 (1) | 1 (4) | 0.287 |
| *SETBP1* | 0 (0) | 0 (0) | 2 (2) | 3 (2) | 7 (5) | 0 (0) | 0.347 |
| *BCOR* | 0 (0) | 1 (2) | 3 (3) | 7 (5) | 10 (8) | 0 (0) | 0.417 |
| *BCORL1* | 0 (0) | 1 (2) | 1 (1) | 2 (1) | 1 (1) | 0 (0) | 0.948 |
| *PHF6* | 0 (0) | 0 (0) | 0 (0) | 0 (0) | 4 (3) | 0 (0) | **0.095** |
| Activated signaling | 1 (25) | 3 (5) | 4 (4) | 8 (5) | 21 (16) | 0 (0) | **0.001** |
| *FLT3-*ITD | 0 (0) | 0 (0) | 1 (0.9) | 0 (0) | 0 (0) | 0 (0) | 0.409 |
| *FLT3-*TKD | 0 (0) | 0 (0) | 0 (0) | 0 (0) | 1 (1) | 0 (0) | 0.687 |
| *KIT* | 0 (0) | 0 (0) | 0 (0) | 0 (0) | 2 (2) | 0 (0) | 0.382 |
| *KRAS* | 0 (0) | 0 (0) | 1 (1) | 1 (1) | 1 (1) | 0 (0) | >0.999 |
| *NRAS* | 0 (0) | 0 (0) | 0 (0) | 3 (2) | 6 (5) | 0 (0) | 0.165 |
| *PTPN11* | 0 (0) | 0 (0) | 0 (0) | 1 (1) | 1 (1) | 0 (0) | >0.999 |
| *JAK2* | 0 (0) | 1 (2) | 1 (0.9) | 1 (1) | 5 (4) | 0 (0) | 0.387 |
| *CBL* | 1 (25) | 1 (2) | 0 (0) | 2 (1) | 4 (3) | 0 (0) | **0.062** |
| *GNAS* | 0 (0) | 0 (0) | 1 (1) | 0 (0) | 1 (1) | 0 (0) | 0.684 |
| Transcription factor | 1 (25) | 2 (3) | 6 (6) | 14 (10) | 36 (28) | 1 (4) | **<0.001** |
| *RUNX1* | 1 (25) | 0 (0) | 5 (5) | 12 (8) | 29 (22) | 0 (0) | **<0.001** |
| *GATA2* | 0 (0) | 1 (2) | 0 (0) | 2 (1.4) | 1 (0.8) | 0 (0) | 0.701 |
| *ETV6* | 0 (0) | 1 (2) | 3 (3) | 0 (0) | 6 (5) | 0 (0) | 0.107 |
| *IKZF1* | 0 (0) | 0 (0) | 0 (0) | 0 (0) | 0 (0) | 1 (4) | **0.057** |
| Spliceosome-complex | 3 (75) | 59 (100) | 14 (13) | 31 (21) | 51 (39) | 4 (17) | **<0.001** |
| *U2AF1* | 1 (25) | 2 (3) | 4 (4) | 10 (7) | 15 (12) | 1 (4) | 0.103 |
| *SRSF2* | 0 (0) | 2 (3) | 6 (6) | 13 (9) | 17 (13) | 1 (4) | 0.242 |
| *ZRSR2* | 0 (0) | 0 (0) | 4 (4) | 5 (3) | 10 (8) | 1 (4) | 0.212 |
| *SF3B1* | 2 (50) | 59 (100) | 0 (0) | 3 (2) | 12 (9) | 1 (4) | **<0.001** |
| Cohesin complex | 1 (25) | 1 (2) | 6 (6) | 13 (9) | 28 (21) | 1 (4) | **<0.001** |
| *RAD21* | 0 (0) | 0 (0) | 0 (0) | 1 (1) | 2 (2) | 0 (0) | 0.800 |
| *SMC1A* | 0 (0) | 0 (0) | 0 (0) | 0 (0) | 1 (1) | 0 (0) | 0.687 |
| *SMC3* | 0 (0) | 0 (0) | 0 (0) | 0 (0) | 1 (1) | 0 (0) | 0.687 |
| *STAG2* | 1 (25) | 1 (2) | 6 (6) | 13 (9) | 24 (18) | 1 (4) | **0.002** |
| Tumor suppressor | 0 (0) | 0 (0) | 0 (0) | 2 (1) | 6 (5) | 23 (100) | **<0.001** |
| *TP53* | 0 (0) | 0 (0) | 0 (0) | 1 (1) | 1 (1) | 23 (100) | **<0.001** |
| *CUX1* | 0 (0) | 0 (0) | 0 (0) | 1 (1) | 5 (4) | 0 (0) | 0.156 |

Data are presented as n (%). *P* values of <0.05 are statistically significant

Abbreviations: SLD, single lineage dysplasia; MLD, multilineage dysplasia; EB, excess blasts; NOS, not otherwise specified

**Supplemental Table 4b.** **Comparison of gene alterations, grouped by functional categories, among patients with myelodysplastic neoplasms, categorized by the 2022 World Health Organization classification**

| Genes | 5q  (n=4) | *SF3B1* (n=62) | LB+RS (n=16) | LB  (n=140) | MDS-h  (n=94) | bi*TP53*  (n=46) | IB1  (n=100) | IB2  (n=149) | MDS-f  (n=24) | *P*  value |
| --- | --- | --- | --- | --- | --- | --- | --- | --- | --- | --- |
| Epigenetics modifiers | 3 (75) | 32 (52) | 5 (31) | 46 (33) | 20 (21) | 16 (35) | 57 (57) | 105 (71) | 12 (50) | **<0.001** |
| DNA methylation | 2 (50) | 26 (42) | 4 (25) | 29 (21) | 9 (10) | 12 (26) | 26 (26) | 57 (38) | 5 (21) | **<0.001** |
| *DNMT3A* | 1 (25) | 10 (16) | 1 (6) | 9 (6) | 2 (2) | 6 (13) | 11 (11) | 17 (11) | 2 (8) | **0.087** |
| *TET2* | 1 (25) | 20 (32) | 2 (13) | 20 (14) | 7 (7) | 7 (15) | 10 (10) | 28 (19) | 0 (0) | **0.001** |
| *IDH1* | 0 (0) | 0 (0) | 0 (0) | 0 (0) | 0 (0) | 0 (0) | 0 (0) | 5 (3) | 0 (0) | 0.115 |
| *IDH2* | 0 (0) | 1 (2) | 2 (13) | 3 (2) | 0 (0) | 0 (0) | 4 (4) | 14 (9) | 3 (13) | **0.001** |
| *WT1* | 0 (0) | 0 (0) | 0 (0) | 2 (1) | 0 (0) | 0 (0) | 4 (4) | 3 (2) | 0 (0) | 0.501 |
| Chromatin modifiers | 1 (25) | 9 (15) | 4 (25) | 26 (19) | 14 (15) | 4 (9) | 45 (45) | 79 (53) | 9 (38) | **<0.001** |
| *ASXL1* | 0 (0) | 6 (10) | 4 (25) | 16 (11) | 10 (11) | 2 (4) | 34 (34) | 57 (38) | 7 (29) | **<0.001** |
| *EZH2* | 1 (25) | 1 (2) | 0 (0) | 3 (2) | 2 (2) | 0 (0) | 7 (7) | 11 (7) | 2 (8) | **0.036** |
| *KMT2A* | 0 (0) | 1 (2) | 0 (0) | 3 (2) | 0 (0) | 2 (4) | 1 (1) | 2 (1) | 0 (0) | 0.624 |
| *SETBP1* | 0 (0) | 0 (0) | 1 (6) | 3 (2) | 1 (1) | 0 (0) | 5 (5) | 8 (5) | 0 (0) | 0.203 |
| *BCOR* | 0 (0) | 2 (3) | 0 (0) | 6 (4) | 3 (3) | 0 (0) | 9 (9) | 15 (10) | 1 (4) | 0.124 |
| *BCORL1* | 0 (0) | 1 (2) | 0 (0) | 1(0.7) | 2 (2) | 0 (0) | 0 (0) | 7 (5) | 1 (4) | 0.177 |
| *PHF6* | 0 (0) | 0 (0.0) | 0 (0) | 0 (0) | 0 (0) | 0 (0) | 4 (4) | 7 (4.7) | 0 (0) | **0.037** |
| Activated signaling | 1 (25) | 3 (5) | 1 (6) | 8 (6) | 2 (2) | 2 (4) | 13 (13) | 30 (20) | 7 (29) | **<0.001** |
| *FLT3-*ITD | 0 (0) | 0 (0) | 0 (0) | 0 (0) | 1 (1) | 1 (2) | 0 (0) | 1 (1) | 0 (0) | 0.459 |
| *FLT3-*TKD | 0 (0) | 0 (0) | 0 (0) | 0 (0) | 0 (0) | 0 (0) | 1 (1) | 2 (1) | 0 (0) | 0.740 |
| *KIT* | 0 (0) | 0 (0) | 0 (0) | 0 (0) | 0 (0) | 0 (0) | 2 (2) | 2 (1) | 0 (0) | 0.547 |
| *KRAS* | 0 (0) | 0 (0) | 0 (0) | 2 (1) | 0 (0) | 0 (0) | 1 (1) | 1 (1) | 0 (0) | 0.926 |
| *NRAS* | 0 (0) | 1 (2) | 0 (0) | 2 (1) | 0 (0) | 0 (0) | 2 (2) | 14 (9) | 1 (4) | **0.004** |
| *PTPN11* | 0 (0) | 0 (0) | 0 (0) | 1 (1) | 0 (0) | 1 (2) | 1 (1) | 4 (3) | 0 (0) | 0.608 |
| *JAK2* | 0 (0) | 1 (2) | 1 (6) | 1 (1) | 0 (0) | 0 (0) | 2 (2) | 2 (1) | 2 (8) | 0.095 |
| *CBL* | 1 (25) | 1 (2) | 0 (0) | 2 (1) | 0 (0) | 0 (0) | 3 (3) | 6 (4) | 1 (4) | **0.097** |
| *GNAS* | 0 (0) | 0 (0) | 0 (0) | 0 (0) | 1 (1) | 0 (0) | 0 (0) | 1 (1) | 0 (0) | 0.822 |
| Transcription factor | 1 (25) | 4 (7) | 2 (13) | 13 (9) | 5 (5) | 6 (13) | 26 (26) | 56 (36) | 3 (13) | **<0.001** |
| *RUNX1* | 1 (25) | 2 (3) | 2 (13) | 9 (6) | 4 (4) | 4 (9) | 23 (23) | 37 (25) | 2 (8) | **<0.001** |
| *GATA2* | 0 (0) | 1 (2) | 0 (0) | 1 (1) | 1 (1) | 0 (0) | 0 (0) | 6 (4) | 0 (0) | 0.346 |
| *ETV6* | 0 (0) | 1 (2) | 0 (0) | 3 (2) | 0 (0) | 0 (0) | 3 (3) | 10 (7) | 1 (4) | 0.120 |
| *IKZF1* | 0 (0) | 0 (0) | 0 (0) | 0 (0) | 0 (0) | 2 (4) | 0 (0) | 3 (2) | 0 (0) | 0.114 |
| Spliceosome-complex | 3 (75) | 62 (100) | 5 (31) | 33 (24) | 4 (4) | 4 (9) | 37 (37) | 64 (43) | 10 (42) | **<0.001** |
| *U2AF1* | 1 (25) | 2 (3) | 1 (6) | 12 (9) | 1 (1) | 0 (0) | 11 (11) | 17 (11) | 5 (21) | **0.003** |
| *SRSF2* | 0 (0) | 2 (3) | 4 (25) | 13 (9) | 2 (2) | 1 (2) | 12 (12) | 26 (17) | 3 (13) | **0.001** |
| *ZRSR2* | 0 (0) | 0 (0) | 0 (0) | 8 (6) | 1 (1) | 3 (7) | 8 (8) | 8 (5) | 1 (4) | 0.177 |
| *SF3B1* | 2 (50) | 62 (100) | 0 (0) | 0 (0) | 0 (0) | 1 (2) | 9 (9) | 15 (10) | 1 (4) | **<0.001** |
| Cohesin complex | 1 (25) | 1 (2) | 1 (6) | 13 (9) | 5 (5) | 2 (4) | 21 (21) | 42 (28) | 2 (8) | **<0.001** |
| *RAD21* | 0 (0) | 0 (0) | 0 (0) | 1 (1) | 0 (0) | 0 (0) | 1 (1) | 1 (1) | 0 (0) | >0.999 |
| *SMC1A* | 0 (0) | 0 (0) | 0 (0) | 0 (0) | 0 (0) | 0 (0) | 1 (1) | 1 (1) | 0 (0) | 0.896 |
| *SMC3* | 0 (0) | 0 (0) | 0 (0) | 0 (0) | 0 (0) | 0 (0) | 1 (1) | 0 (0) | 0 (0) | 0.545 |
| *STAG2* | 1 (25) | 1 (2) | 1 (6) | 13 (9) | 5 (5) | 2 (4) | 18 (18) | 40 (27) | 2 (8) | **<0.001** |
| Tumor suppressor | 0 (0) | 0 (0) | 0 (0) | 2 (1) | 0 (0) | 46 (100) | 5 (5) | 12 (8) | 5 (21) | **<0.001** |
| *TP53* | 0 (0) | 0 (0) | 0 (0) | 1 (1) | 0 (0) | 46 (100) | 1 (1) | 11 (7) | 4 (17) | **<0.001** |
| *CUX1* | 0 (0) | 0 (0) | 0 (0) | 1 (1) | 0 (0) | 0 (0) | 4 (4) | 1 (1) | 1 (4) | 0.157 |

Data are presented as n (%).

*P* values of <0.05 are statistically significant

Abbreviations: 5q, MDS with low blasts and isolated 5q deletion; bi*TP53*, MDS with biallelic *TP53* inactivation; MDS-f, MDS with fibrosis; MDS-h, hypoplastic MDS; IB1, MDS with increased blasts-1; IB2, MDS with increased blasts-2; LB, MDS with low blasts; RS, ring sideroblasts; *SF3B1*, MDS with low blasts and *SF3B1* mutation

**Supplemental Table 4c. Comparison of gene alterations, grouped by functional categories, among patients with different subtypes of myelodysplastic syndromes/acute myeloid leukemia, categorized by the 2022 International Consensus Classification**

| Genes | Mutated *TP53*  (n=37) | MDS-related gene mutations  (n=93) | MDS-related cytogenetics  (n=12) | NOS  (n=20) | *P* value |
| --- | --- | --- | --- | --- | --- |
| Epigenetics modifiers | 10 (27) | 75 (81) | 6 (50) | 12 (60) | **<0.001** |
| DNA methylation | 6 (16) | 37 (40) | 5 (42) | 11 (55) | **0.016** |
| *DNMT3A* | 4 (11) | 9 (10) | 2 (17) | 5 (25) | 0.249 |
| *TET2* | 2 (5) | 20 (22) | 2 (17) | 3 (15) | 0.149 |
| *IDH1* | 0 (0) | 3 (3) | 1 (8) | 1 (5) | 0.249 |
| *IDH2* | 0 (0) | 11 (12) | 1 (8) | 2 (10) | 0.104 |
| *WT1* | 0 (0) | 0 (0) | 0 (0) | 2 (10) | **0.020** |
| Chromatin modifiers | 5 (14) | 65 (70) | 1 (8) | 1 (5) | **<0.001** |
| *ASXL1* | 4 (11) | 49 (53) | 0 (0) | 0 (0) | **<0.001** |
| *EZH2* | 0 (0) | 10 (11) | 0 (0) | 0 (0) | **0.024** |
| *MLL* | 1 (3) | 1 (1) | 1 (8) | 0 (0) | 0.201 |
| *SETBP1* | 0 (0) | 6 (7) | 0 (0) | 0 (0) | 0.379 |
| *BCOR* | 0 (0) | 14 (15) | 0 (0) | 0 (0) | **0.009** |
| *BCORL1* | 0 (0) | 7 (8) | 0 (0) | 0 (0) | 0.267 |
| *PHF6* | 0 (0) | 6 (7) | 0 (0) | 1 (5) | 0.386 |
| Activated signaling | 3 (8) | 22 (24) | 0 (0) | 3 (15) | **0.060** |
| *FLT3-*ITD | 1 (3) | 0 (0) | 0 (0) | 0 (0) | 0.426 |
| *FLT3-*TKD | 0 (0) | 1 (1) | 0 (0) | 1 (5) | 0.408 |
| *KIT* | 1 (3) | 1 (1) | 0 (0) | 0 (0) | 0.672 |
| *KRAS* | 0 (0) | 1 (1) | 0 (0) | 0 (0) | >0.999 |
| *NRAS* | 0 (0) | 10 (19) | 0 (0) | 1 (5) | 0.137 |
| *PTPN11* | 1 (3) | 4 (4) | 0 (0) | 0 (0) | >0.999 |
| *JAK2* | 0 (0) | 1 (1) | 0 (0) | 0 (0) | >0.999 |
| *CBL* | 0 (0) | 5 (5) | 0 (0) | 1 (5) | 0.554 |
| *GNAS* | 0 (0) | 0 (0) | 0 (0) | 0 (0) | - |
| Transcription factor | 5 (14) | 44 (47 | 0 (0) | 4 (20) | **<0.001** |
| *RUNX1* | 4 (11) | 32 (34) | 0 (0) | 0 (0) | **<0.001** |
| *GATA2* | 0 (0) | 4 (4) | 0 (0) | 1 (5) | 0.668 |
| *ETV6* | 0 (0) | 7 (8) | 0 (0) | 1 (5) | 0.318 |
| *IKZF1* | 1 (3) | 1 (1) | 0 (0) | 2 (10) | **0.090** |
| Spliceosome-complex | 3 (8) | 57 (61) | 0 (0) | 0 (0) | **<0.001** |
| *U2AF1* | 0 (0) | 17 (18) | 0 (0) | 0 (0) | **0.002** |
| *SRSF2* | 2 (5) | 22 (24) | 0 (0) | 0 (0) | **0.003** |
| *ZRSR2* | 2 (5) | 7 (8) | 0 (0) | 0 (0) | 0.722 |
| *SF3B1* | 0 (0) | 13 (14) | 0 (0) | 0 (0) | **0.017** |
| Cohesin complex | 3 (8) | 34 (37) | 0 (0) | 1 (5) | **<0.001** |
| *RAD21* | 0 (0) | 0 (0) | 0 (0) | 0 (0) | - |
| *SMC1A* | 0 (0) | 0 (0) | 0 (0) | 1 (5) | 0.198 |
| *SMC3* | 0 (0) | 0 (0) | 0 (0) | 0 (0) | - |
| *STAG2* | 3 (8) | 34 (37) | 0 (0) | 0 (0) | **<0.001** |
| Tumor suppressor | 37 (100) | 2 (2) | 0 (0) | 0 (0) | **<0.001** |
| *TP53* | 37 (100) | 1 (1) | 0 (0) | 0 (0) | **<0.001** |
| *CUX1* | 0 (0) | 1 (1) | 0 (0) | 0 (0) | >0.999 |

Data are presented as n (%). *P* values of <0.05 are statistically significant

Abbreviations: NOS, not otherwise specified

**Supplemental Table 5. Time-dependent Cox regression subgroup analysis (adjusted for age) for the impact of allogeneic hematopoietic stem cell transplantation on leukemia-free survival and overall survival.**

| ICC | LFS | | OS | |
| --- | --- | --- | --- | --- |
|  | **HR (95% CI)** | ***P* value** | **HR (95% CI)** | ***P* value** |
| MDS with mutated *SF3B1* | 0.685 (0.074-6.318) | 0.738 | 1.743 (0.292-10.392) | 0.542 |
| MDS, NOS with SLD | 2.096 (0.243-18.040) | 0.501 | 4.553 (0.879-23.586) | 0.071 |
| MDS, NOS with MLD | 0.997 (0.78-3.580) | 0.997 | 1.440 (0.456-4.543) | 0.534 |
| MDS with EB | 0.419 (0.202-0.867) | **0.019** | 0.735 (0.367-1.472) | 0.385 |
| MDS with mutated *TP53* | 0.793 (0.070-7.612) | 0.793 | 0.596 (0.060-5.884) | 0.658 |
| MDS/AML with mutated *TP53* | 0.147 (0.011-1.939) | 0.145 | 0.601 (0.088-4.131) | 0.605 |
| MDS/AML with MDS-related genes | 0.546 (0.224-1.329) | 0.182 | 0.989 (0.448-2.182) | 0.978 |
| MDS/AML with MDS-related cytogenetics | 0.633 (0.064-6.233) | 0.695 | 0.521 (0.049-5.506) | 0.588 |
| MDS/AML, NOS | 0.901 (0.129-6.290) | 0.916 | 2.067 (0.275-15.538) | 0.480 |

*P* values of <0.05 are statistically significant.

Abbreviations: AML, acute myeloid leukemia; CI, confidence interval; EB, excess blasts; HR, hazard ratios; ICC, International Consensus Classification; LFS, leukemia-free survival; MDS, myelodysplastic syndromes; NOS, not otherwise specified; OS, overall survival

**Supplemental Table 6. Time-dependent Cox regression subgroup analysis for the impact of allogeneic hematopoietic stem cell transplantation on leukemia-free survival and overall survival.**

| WHO-2022 classification | LFS | | OS | |
| --- | --- | --- | --- | --- |
|  | **HR (95% CI)** | ***P* value** | **HR (95% CI)** | ***P* value** |
| MDS-*SF3B1* | 0.674 (0.075-6.093) | 0.725 | 1.681 (0.293-9.643) | 0.560 |
| MDS-LB | 0.980 (0.219-4.389) | 0.979 | 2.239 (0.722-6.947) | 0.163 |
| MDS-h | 1.745 (0.305-9.990) | 0.532 | 1.717 (0.300-9.813) | 0.543 |
| MDS-IB1 | 0.450 (0.188-1.079) | **0.074** | 0.835 (0.375-1.858) | 0.658 |
| MDS-IB2 | 0.597 (0.285-1.248) | 0.170 | 0.988 (0.489-1.995) | 0.973 |
| MDS-f | 0.324 (0.074-1.430) | 0.137 | 0.476 (0.119-1.902) | 0.294 |
| MDS-bi*TP53* | 0.436 (0.05-2.549) | 0.357 | 1.310 (0.328-5.237) | 0.702 |

*P* values of <0.05 are statistically significant.

Abbreviations: bi*TP53*, biallelic *TP53* inactivation; CI, confidence interval; f, fibrosis; h, hypoplastic; HR, hazard ratios; IB1, increased blasts-1; IB2, increased blasts-2; LB, low blasts; LFS, leukemia-free survival; MDS, myelodysplastic neoplasms; OS, overall survival; WHO, World Health Organization

**Supplemental Table 7. Case allocation from the 2022 International Consensus Classification to the 2022 World Health Organization classification of 635 patients with myelodysplastic syndromes/neoplasms**

| WHO-2022 | n=635 | ICC | n (%) |
| --- | --- | --- | --- |
| MDS with low blasts and isolated 5q deletion | 4 | **MDS with del(5q)** | 4 (100) |
| MDS with low blasts and *SF3B1* mutation | 62 | **MDS with mutated *SF3B1*** | 59 (95.2) |
|  |  | **MDS, NOS, with MLD** | 3 (4.8) |
| MDS with low blasts and RS | 16 | **MDS, NOS, with SLD** | 9 (56.3) |
|  |  | **MDS, NOS, with MLD** | 7 (43.8) |
| MDS with biallelic *TP53* inactivation | 46 | **MDS with mutated *TP53*** | 17 (37.0) |
|  |  | **MDS/AML with mutated *TP53*** | 29 (63.0) |
| MDS with low blasts | 140 | **MDS, NOS, with SLD** | 58 (41.4) |
|  |  | **MDS, NOS, with MLD** | 82 (58.6) |
| MDS, hypoplastic | 94 | **MDS, NOS, with SLD** | 39 (41.5) |
|  |  | **MDS, NOS, with MLD** | 55 (58.5) |
| MDS with increased blasts-1 | 100 | **MDS with EB** | 99 (99.0) |
|  |  | **MDS with mutated *TP53*** | 1 (1.0) |
| MDS with increased blasts-2 | 149 | **MDS with EB** | 19 (12.8) |
|  |  | **MDS with mutated *TP53*** | 1 (0.7) |
|  |  | **MDS/AML with mutated *TP53*** | 8 (5.4) |
|  |  | **MDS/AML with MDS-related gene*** | 87 (58.4) |
|  |  | **MDS/AML with MDS-related cytogenetics^†^** | 12 (8.1) |
|  |  | **MDS/AML, NOS** | 19 (12.8) |
|  |  | **AML with *CEBPA*** | 3 (2.0) |
| MDS with fibrosis | 24 | **MDS with EB** | 13 (54.2) |
|  |  | **MDS with mutated *TP53*** | 4 (16.7) |
|  |  | **MDS/AML with MDS-related gene*** | 6 (25.0) |
|  |  | **MDS/AML, NOS** | 1 (4.2) |

Abbreviations: AML, acute myeloid leukemia; EB, excess blasts; ICC, International Consensus Classification; MDS, myelodysplastic syndromes/neoplasms; MLD, multilineage dysplasia; NOS, not otherwise specified; RS, ring sideroblasts; SLD, single lineage dysplasia; WHO, World Health Organization

*MDS-related gene mutations: *ASXL1, BCOR, EZH2, RUNX1, SF3B1, SRSF2, STAG2, U2AF1*, or *ZRSR2*

^†^MDS-related cytogenetic abnormalities: complex (>3 clones) karyotype (in the absence of a *TP53* mutation), del(5q)/t(5q)/add(5q), -7/del(7q), +8, del(12p)/t(12p)/add(12p), i(17q), -17/add(17p) or del(17p), del(20q), and/or idic(X)(q13) clonal abnormalities

**Supplemental Table 8. Univariable Cox regression analysis for leukemia-free survival and overall survival.**

| Variables | LFS | | OS | |
| --- | --- | --- | --- | --- |
|  | **HR (95% CI)** | ***P* value** | **HR (95% CI)** | ***P* value** |
| Age^*^ | 1.026 (1.018-1.033) | **<0.001** | 1.028 (1.020-1.036) | **<0.001** |
| Female | 0.668 (0.527-0.848) | **0.001** | 0.659 (0.518-0.838) | **0.001** |
| Refined system |  | **<0.001** |  | **<0.001** |
| MDS-h, SLD | Reference | - | Reference | - |
| Low-risk MDS^†^ | 1.352 (0.925-1.977) | 0.120 | 1.355 (0.925-1.985) | 0.119 |
| MDS with EB^‡^ | 2.837 (1.964-4.099) | **<0.001** | 2.727 (1.887-3.941) | **<0.001** |
| MDS/AML^§^ | 5.322 (3.760-7.532) | **<0.001** | 4.521 (3.179-6.429) | **<0.001** |
| Mutated *TP53*^¶^ | 17.343 (11.237-26.767) | **<0.001** | 20.664 (13.267-32.187) | **<0.001** |
| IPSS-M |  | **<0.001** |  | **<0.001** |
| Very low/low | Reference | - | Reference | - |
| Moderate low | 1.856 (1.156-2.982) | **0.011** | 1.869 (1.164-3.003) | **0.010** |
| Moderate high | 2.576 (1.656-4.005) | **<0.001** | 2.382 (1.520-3.732) | **<0.001** |
| High | 3.949 (2.644-5.899) | **<0.001** | 3.921 (2.625-5.857) | **<0.001** |
| Very high | 11.304 (7.732-16.526) | **<0.001** | 10.238 (6.993-14.990) | **<0.001** |
| IPSS-R |  | **<0.001** |  | **<0.001** |
| Very low/low | Reference | - | Reference | - |
| Intermediate | 1.754 (1.248-2.465) | **0.001** | 1.663 (1.179-2.345) | **0.004** |
| High | 3.557 (2.557-4.947) | **<0.001** | 3.410 (2.449-4.749) | **<0.001** |
| Very high | 6.259 (4.519-8.667) | **<0.001** | 6.028 (4.340-8.373) | **<0.001** |
| HSCT | 0.790 (0.549-1.135) | 0.202 | 1.080 (0.792-1.473) | 0.627 |

Note: Only 17 patients (2.7%) were categorized as IPSS-M very low risk and there was no inter-group difference between IPSS-M very low and low risk subgroups in both OS and LFS; accordingly, we put IPSS-M very low and low groups together.

*P* values of <0.05 are statistically significant.

*As continuous variables analysis.

^†^Low-risk MDS included MDS with del(5q), MDS with mutated *SF3B1*, MDS, NOS with MLD

^‡^MDS patients with EB and blast percentage < 5% in peripheral blood.

^§^MDS/AML with MDS-related gene mutations, MDS-related cytogenetic abnormalities, or not otherwise specified. Patients with MDS with EB and blast percentage ≥ 5% in peripheral blood were included in this group.

^¶^MDS or MDS/AML with mutated *TP53*

Abbreviations: CI, confidence interval; HR, hazard ratio; HSCT, allogeneic hematopoietic stem cell transplantation; h, hypoplastic; IPSS-M, Molecular International Prognostic Scoring System; IPSS-R, Revised International Prognostic Scoring System; LFS, leukemia-free survival; MDS, myelodysplastic neoplasm; OS, overall survival.

**Supplemental Figure 1. Distribution of IPSS-R and IPSS-M based on the 2022 World Health Organization classification or International Consensus Classification of myelodysplastic syndromes/neoplasms**

(a) Distribution of IPSS-R based on the International Consensus Classification

(b) Distribution of IPSS-M based on the International Consensus Classification

(c) Distribution of IPSS-R based on the 2022 World Health Organization classification

(d) Distribution of IPSS-M based on the 2022 World Health Organization classification

| (a) |
| --- |
|  |
| (b) |
|  |
|  |
|  |
|  |
|  |
|  |
|  |
|  |
| (c) |
| **MDS-bi*TP53***  **MDS-*SF3B1*** |
| (d) |
| **MDS-*SF3B1***  **MDS-bi*TP53*** |
|  |

Abbreviations: EB, excess blasts; IPSS-R, Revised International Prognosis Scoring System; IPSS-M, Molecular International Prognosis Scoring System; MDS, myelodysplastic syndromes/neoplasms; MDS-5q, MDS with low blasts and isolated 5q deletion; MDS-LB and RS, MDS with low blasts and ring sideroblasts; MDS-LB, MDS with low blasts; MDS-h, hypoplastic MDS; MDS-IB1, MDS with increased blasts-1; MDS-IB2, MDS with increased blasts-2; MDS-f, MDS with fibrosis; MDS-bi*TP53*, MDS with biallelic *TP53* inactivation; MLD, multilineage dysplasia; NOS, not otherwise specified; SLD, single lineage dysplasia

**Supplemental Figure 2. Overview of mutations in each subtype of myelodysplastic syndromes (MDS), or MDS/acute myeloid leukemia (AML) based on the International Consensus Classification**

(a) Genetic profiles of MDS with del(5q)

(b) Genetic profiles of MDS with mutated *SF3B1*

(c) Genetic profiles of MDS, not otherwise specified with single lineage dysplasia (NOS, with SLD)

(d) Genetic profiles of MDS, NOS, with multilineage dysplasia (MLD)

(e) Genetic profiles of MDS with excess blasts (EB)

(f) Genetic profiles of MDS with mutated *TP53*

(g) Genetic profiles of MDS/AML with mutated *TP53*

(h) Genetic profiles of MDS/AML with myelodysplasia-related gene mutations

(i) Genetic profiles of MDS/AML with myelodysplasia-related cytogenetics abnormalities

(j) Genetic profiles of MDS/AML, NOS

| (a) MDS with del(5q) | (b) MDS with mutated *SF3B1* |
| --- | --- |
|  |  |

| (c) MDS, NOS with SLD |  |
| --- | --- |
|  |  |
|  |  |
| (d) MDS, NOS with MLD |  |
|  |  |

| (e) MDS with EB | | | (f) MDS with mutated *TP53* |
| --- | --- | --- | --- |
|  | | |  |
|  |  |  | |
| (g) MDS/AML with mutated *TP53* | (h) MDS/AML with MDS-related genes mutations | (i) MDS/AML with MDS-related cytogenetic | |
|  |  |  | |

| (j) MDS/AML, NOS |  |
| --- | --- |
|  |  |

Note: Each column represents an individual patient, and each color represent an individual mutation

**Supplemental Figure 3. Overview of mutations in each subtype of myelodysplastic neoplasms (MDS) based on the 2022 World Health Organization classification**

(a) Genetic profiles of MDS with low blasts and isolated 5q deletion (MDS-5q)

(b) Genetic profiles of MDS with low blasts and *SF3B1* mutation (MDS-*SF3B1*)

(c) Genetic profiles of MDS with low blasts and ring sideroblasts (MDS-LB and RS)

(d) Genetic profiles of MDS with low blasts (MDS-LB)

(e) Genetic profiles of MDS, hypoplastic (MDS-h)

(f) Genetic profiles of MDS with increased blasts-1 (MDS-IB1)

(g) Genetic profiles of MDS with increased blasts-2 (MDS-IB2)

(h) Genetic profiles of MDS with fibrosis (MDS-f)

(i) Genetic profiles of MDS with biallelic *TP53* inactivation (MDS-bi*TP53*)

| (a) MDS-5q | (b) MDS-*SF3B1* | (c) MDS-LB and RS |
| --- | --- | --- |
| **** | **** |  |
| (d) MDS-LB | | |
| **** | | |
|  | | |
| (e) MDS-h | | |
|  | | |

| (f) MDS-IB1 |  |
| --- | --- |
| **** |  |
|  |  |
| (g) MDS-IB2 |  |
| **** |  |

| (h) MDS-f | (i) MDS-bi*TP53* |
| --- | --- |
| **** |  |

Note: Each column represents an individual patient, and each color represent an individual mutation

**Supplemental Figure 4. Kaplan-Meier curves for leukemia-free survival and overall survival in patients with myelodysplastic syndromes based on the International Consensus Classification**

(a) Leukemia-free survival for patients with myelodysplastic syndromes

(b) Overall survival for patients with myelodysplastic syndromes

(c) Leukemia-free survival for patients with myelodysplastic syndromes/acute myeloid leukemia

(d) Overall survival for patients with myelodysplastic syndromes/acute myeloid leukemia

| (a) | (b) |
| --- | --- |
| 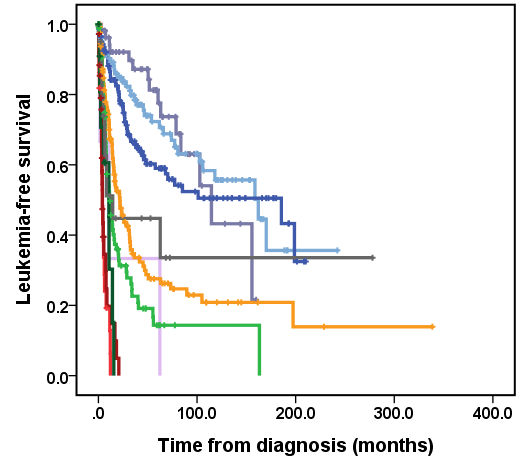  ***P*<0.001** | 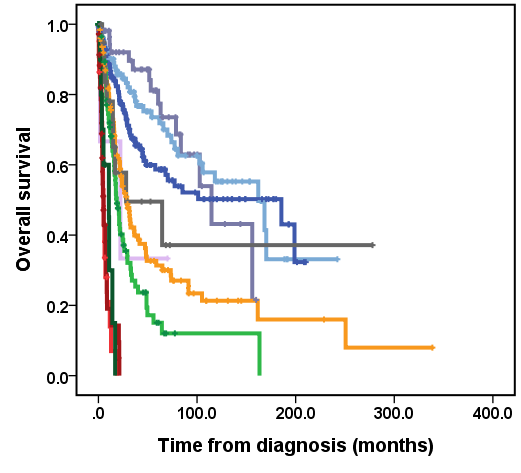  ***P*<0.001** |

**MDS with mutated *SF3B1***, n=59

**MDS with del(5q)**, n=4

**MDS, NOS with MLD**, n=147

**MDS with EB**, n=131

**MDS with mutated *TP53***, n=23

**MDS/AML with MDS-related cytogenetics abnormalities**, n=12

**MDS/AML with mutated *TP53***, n=37

**MDS/AML with MDS-related gene mutations**, n=93

**MDS, NOS with SLD**, n=106

**MDS/AML, NOS**, n=20

Abbreviations: AML, acute myeloid leukemia; EB, excess blasts; MDS, myelodysplastic syndromes; MLD, multilineage dysplasia; NOS, not otherwise specified; SLD, single lineage dysplasia

**Supplemental Figure 5.** **Kaplan-Meier curves for leukemia-free survival and overall survival in patients with myelodysplastic neoplasms with fibrosis, stratified by different blast ranges.**

(a) Leukemia-free survival for patients with peripheral blood (PB) blast 2-4%, or bone marrow (BM) blast 5-9%

(b) Overall survival for patients with PB blast 5-19%, or BM blast 10-19%

| (a) | (b) |
| --- | --- |
| 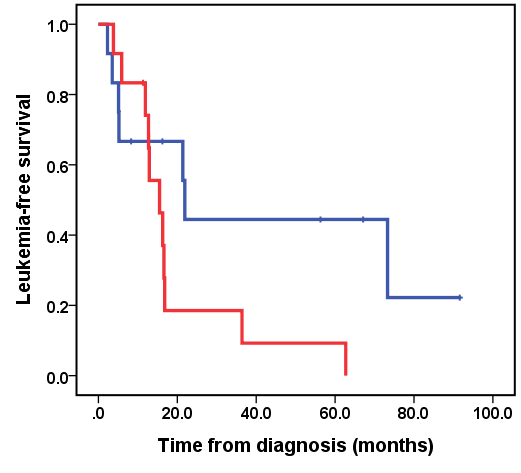  **PB blast 2-4%, or BM blast 5-9%**, n=12  **PB blast 5-19%, or BM blast 10-19%**, n=12  ***P*=0.087** | 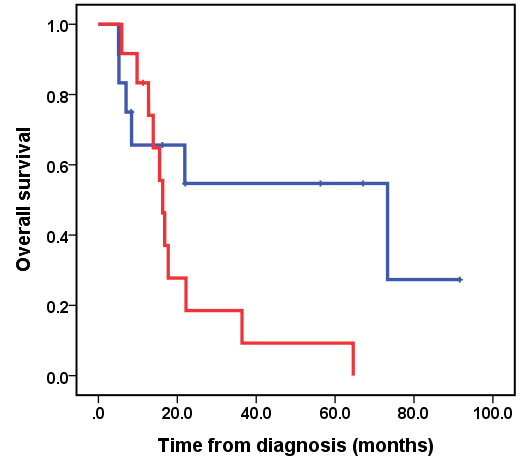  **PB blast 5-19%, or BM blast 10-19%**, n=12  **PB blast 2-4%, or BM blast 5-9%**, n=12  ***P*=0.081** |

**Supplemental Figure 6. Kaplan-Meier curves for leukemia-free survival and overall survival in patients with myelodysplastic syndromes (MDS), not otherwise specified with single/multilineage dysplasia, classified according to the 2022 World Health Organization (WHO) classification**

(a) Leukemia-free survival for patients with hypoplastic MDS (MDS-h) and MDS with low blasts (MDS-LB), classified according to the 2022 WHO classification

(b) Overall survival for patients with MDS-h and MDS-LB, classified according to the 2022 WHO classification

| (a) | (b) |
| --- | --- |
| 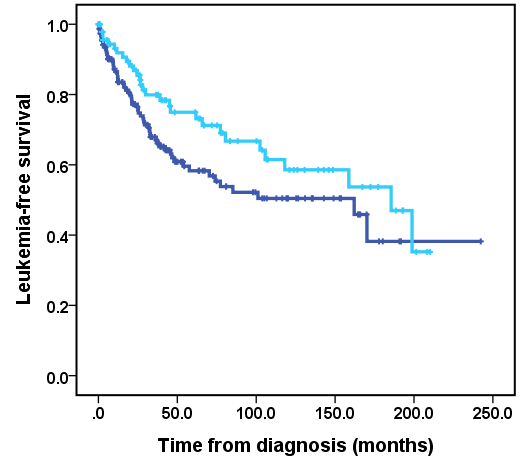  ***P*=0.075**  **MDS-h,** n=94  **MDS-LB,** n=156 | 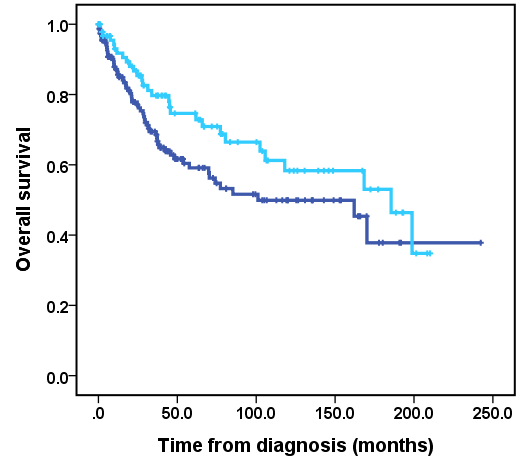  **MDS-LB,** n=156  **MDS-h,** n=94  ***P*=0.070** |

**Supplemental Figure 7. Kaplan-Meier curves for leukemia-free survival and overall survival in patients with myelodysplastic syndromes (MDS), with excess blasts, classified according to the 2022 World Health Organization (WHO) classification**

(a) Leukemia-free survival for patients with MDS with increased blasts-1 (MDS-IB1), MDS with increased blasts-2 (MDS-IB2) and MDS with fibrosis (MDS-f), classified according to the 2022 WHO classification

(b) Overall survival for patients with MDS-IB1, MDS-IB2 and MDS-f, classified according to the 2022 WHO classification

| (a) | (b) |
| --- | --- |
| 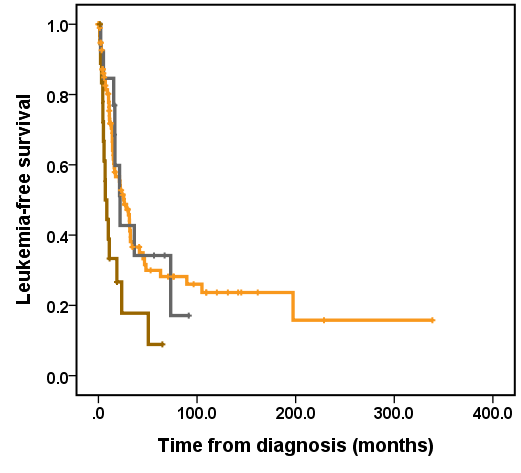  **MDS-IB2**, n=19  **MDS-f**, n=13  **MDS-IB1**, n=99  ***P*=0.004**  ***P*=0.042**  ***P*=0.883** | 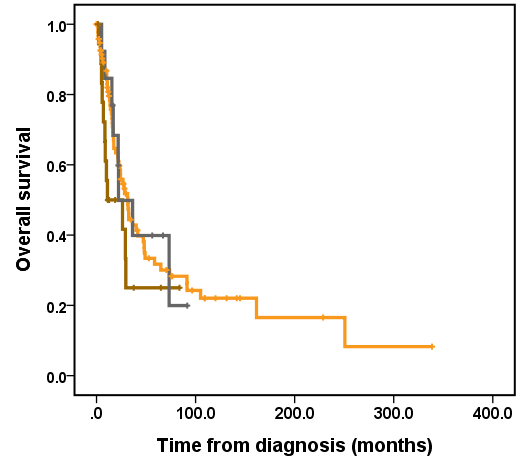  **MDS-IB2**, n=19  **MDS-f**, n=13  **MDS-IB1**, n=99  ***P*=0.123**  ***P*=0.333**  ***P*=0.887** |

**Supplemental Figure 8. Kaplan-Meier curves for leukemia-free survival and overall survival in patients with myelodysplastic syndromes (MDS) with mutated *TP53*, classified according to the 2022 World Health Organization (WHO) classification**

(a) Leukemia-free survival for patients with MDS with biallelic *TP53* inactivation (MDS-bi*TP53*), MDS with increased blasts (MDS-IB), and MDS with fibrosis (MDS-f), classified according to the 2022 WHO classification

(b) Overall survival for patients with MDS-bi*TP53*, MDS-IB, and MDS-f, classified according to the 2022 WHO classification

| (a) | (b) |
| --- | --- |
| 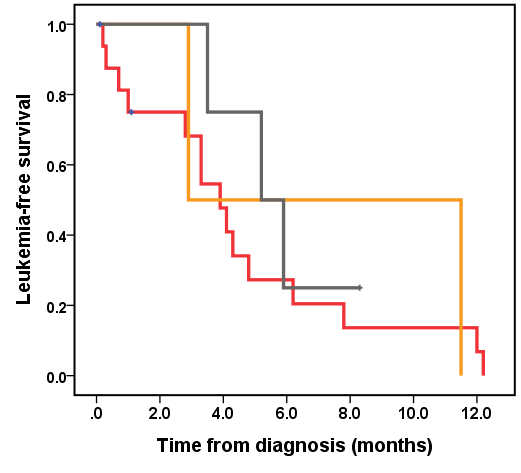  **MDS-IB**, n=2  **MDS-f**, n=4  **MDS-bi*TP53***, n=17  ***P*=0.817**  ***P*=0.896**  ***P*=0.388** | 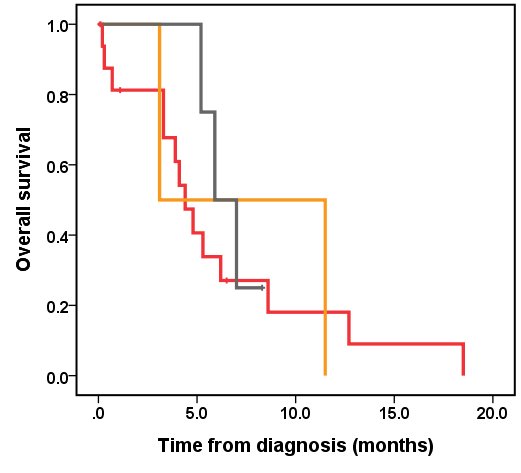  **MDS-IB**, n=2  **MDS-f**, n=4  **MDS-bi*TP53***, n=17  ***P*=0.981**  ***P*=0.896**  ***P*=0.472** |

**Supplemental Figure 9. Kaplan-Meier curves for leukemia-free survival and overall survival in patients with myelodysplastic syndromes/acute myeloid leukemia (MDS/AML) with MDS-related gene mutations, classified according to the 2022 World Health Organization classification**

(a) Leukemia-free survival for patients with MDS with increased blasts-2 (MDS-IB2), and MDS with fibrosis (MDS-f), classified according to the 2022 WHO classification

(b) Overall survival for patients with MDS-IB2, and MDS-f, classified according to the 2022 WHO classification

| (a) | (b) |
| --- | --- |
| 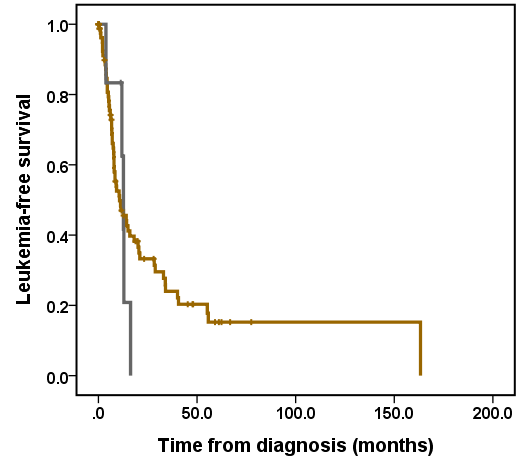  ***P*=0.630**  **MDS-f,** n=6  **MDS-IB2,** n=87 | 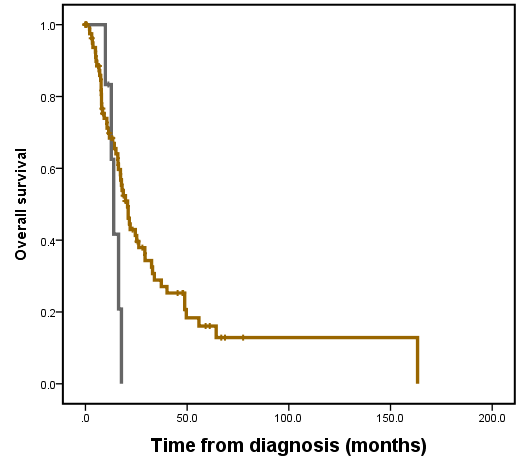  ***P*=0.126**  **MDS-f,** n=6  **MDS-IB2,** n=87 |

**Supplemental Figure 10.** **Kaplan-Meier curves for leukemia-free survival and overall survival in patients with myelodysplastic neoplasms (MDS) with low blasts (further classified according to the International Consensus Classification [ICC]), and hypoplastic MDS**

(a) Leukemia-free survival for patients with MDS, not otherwise specified with single lineage dysplasia (MDS, NOS with SLD), MDS, NOS with multilineage dysplasia (MLD), and hypoplastic MDS (MDS-h)

(b) Overall survival for patients with myelodysplastic syndromes for patients with MDS, NOS with SLD, MDS, NOS with MLD, and MDS-h

| (a) | (b) |
| --- | --- |
| 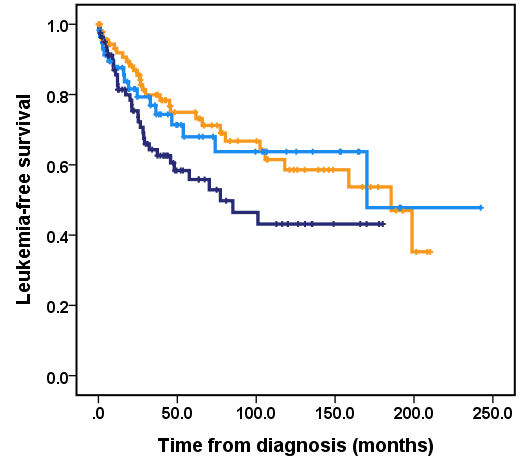  **MDS, NOS with SLD**, n=58  **MDS, NOS with MLD**, n=82  **MDS-h**, n=94  ***P*=0.828**  ***P*=0.153**  ***P*=0.037** | 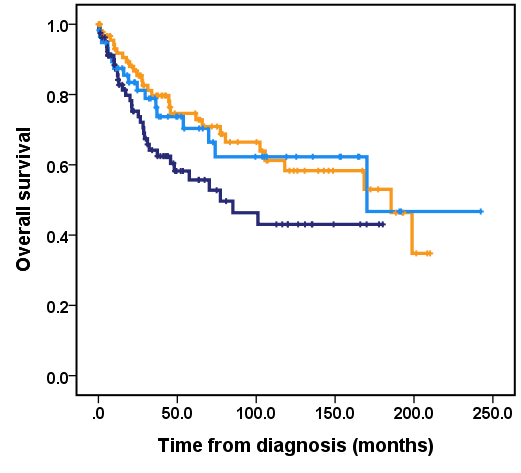  **MDS, NOS with SLD**, n=58  **MDS, NOS with MLD**, n=82  **MDS-h**, n=94  ***P*=0.824**  ***P*=0.140**  ***P*=0.034** |

**Supplemental Figure 11. Kaplan-Meier curves for leukemia-free survival and overall survival in patients with myelodysplastic neoplasms (MDS) with biallelic *TP53* inactivation, classified according to the International Consensus Classification (ICC)**

(a) Leukemia-free survival for patients with MDS/acute myeloid leukemia (AML) with mutated *TP53* and MDS with mutated *TP53*, classified according to the ICC

(b) Overall survival for patients with MDS/AML with mutated *TP53* and MDS with mutated *TP53*, classified according to the ICC

| (a) | (b) |
| --- | --- |
| 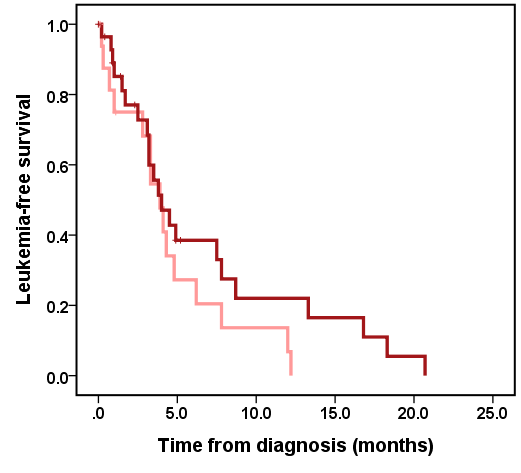  **MDS with mutated *TP53*,** n=17  ***P*=0.211**  **MDS/AML with mutated *TP53*,** n=29 | 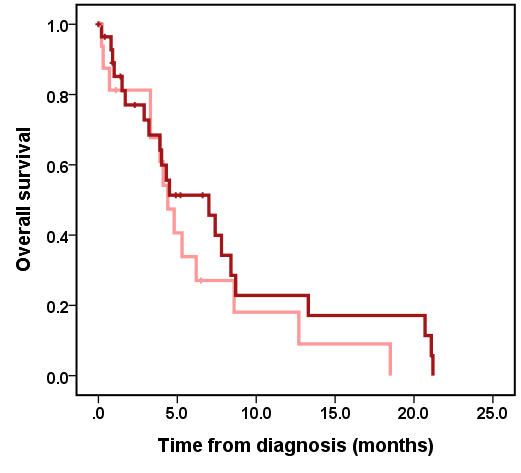  **MDS/AML with mutated *TP53*,** n=29  **MDS with mutated *TP53*,** n=17  ***P*=0.293** |

**Supplemental Figure 12. Kaplan-Meier curves for leukemia-free survival and overall survival in patients with myelodysplastic neoplasms (MDS) with increased blasts-2, classified according to the International Consensus Classification (ICC)**

(a) Leukemia-free survival for patients with MDS/acute myeloid leukemia (AML), not otherwise specified (NOS), MDS/AML with MDS-related gene mutations, MDS with excess blasts (EB), MDS/AML with MDS-related cytogenetics abnormalities, and MDS/AML with mutated *TP53*, classified according to the ICC

(b) Overall survival for patients with MDS/AML-NOS, MDS/AML with MDS-related gene mutations, MDS with EB, MDS/AML with MDS-related cytogenetics abnormalities, and MDS/AML with mutated *TP53*, classified according to the ICC

| (a) |
| --- |
| 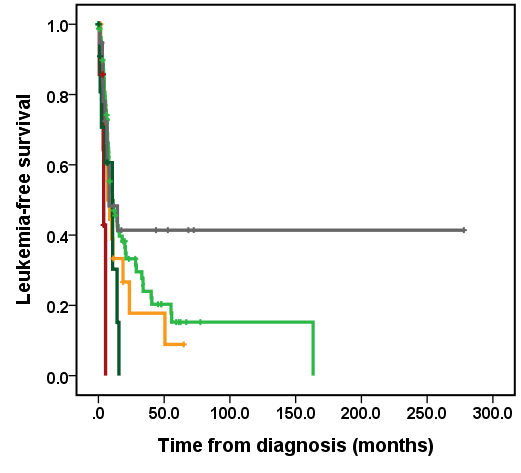  **MDS with EB**, n=19  **MDS/AML with MDS-related cytogenetics abnormalities**, n=12  **MDS/AML with mutated *TP53***, n=8  **MDS/AML with MDS-related gene mutations**, n=87  **MDS/AML-NOS**, n=19  ***P*=0.043**  ***P*=0.004**  ***P*=0.052**  ***P*=0.301**  ***P*=0.310**  ***P*=0.399**  ***P*=0.240** |
| (b) |
| 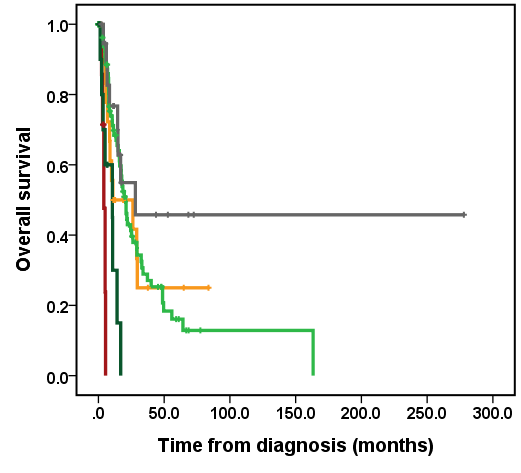  **MDS with EB**, n=19  **MDS/AML with MDS-related cytogenetics abnormalities**, n=12  **MDS/AML with mutated *TP53***, n=8  **MDS/AML with MDS-related gene mutations**, n=87  **MDS/AML-NOS**, n=19  ***P*<0.001**  ***P*<0.001**  ***P*<0.001**  ***P*=0.123**  ***P*=0.864**  ***P*=0.131**  ***P*=0.058** |

**Supplemental Figure 13. Kaplan-Meier curves for leukemia-free survival and overall survival in patients with myelodysplastic neoplasms (MDS) with fibrosis, classified according to the International Consensus Classification (ICC)**

(a) Leukemia-free survival for patients with MDS with excess blasts (EB), MDS/acute myeloid leukemia (AML) with MDS-related gene mutations, and MDS with mutated *TP53*, classified according to the ICC

(b) Overall survival for patients with MDS with EB, MDS/AML with MDS-related gene mutations, and MDS with mutated *TP53*, classified according to the ICC

| (a) |
| --- |
| 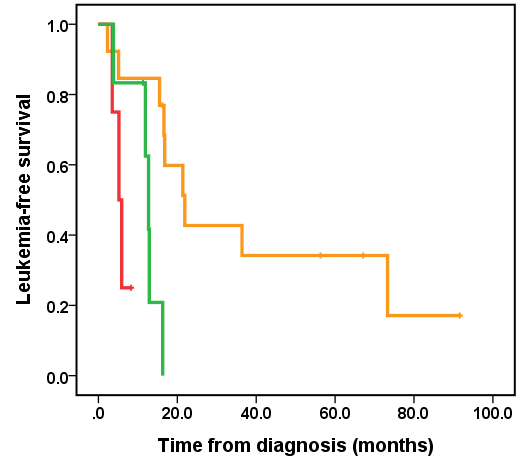  **MDS/AML with MDS-related gene mutations**, n=6  **MDS with mutated *TP53***, n=4  **MDS with EB**, n=13  ***P*=0.005**  ***P*=0.091**  ***P*=0.032** |
| (b) |
| 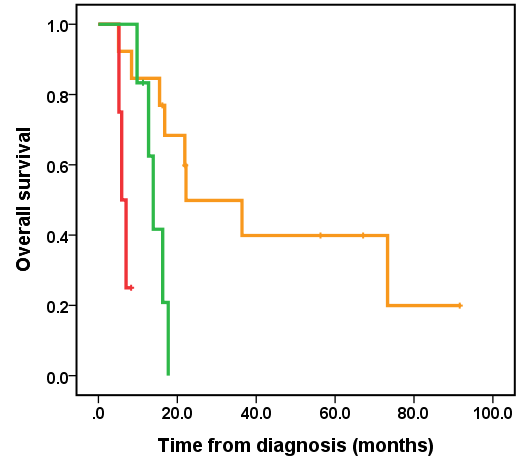  **MDS/AML with MDS-related gene mutations**, n=6  **MDS with mutated *TP53***, n=4  **MDS with EB**, n=13  ***P*=0.015**  ***P*=0.012**  ***P*=0.007** |
